# Maternal administration of octanoate, a medium-chain fatty acid, improves feed efficiency of Japanese black calves through influencing gut bacteriome structure

**DOI:** 10.1038/s41598-025-18490-0

**Published:** 2025-09-29

**Authors:** Haruki Yamano, Hiroshi Horike, Yutaka Taguchi, Yudai Inabu, Hirokuni Miyamoto, Atsushi Kurotani, Nonomi Suzuki, Shigeharu Moriya, Teruno Nakaguma, Chitose Ishii, Makiko Matsuura, Naoko Tsuji, Tetsuji Etoh, Yuji Shiotsuka, Ryoichi Fujino, Satoshi Wada, Jun Kikuchi, Hiroshi Ohno, Hideyuki Takahashi

**Affiliations:** 1 Kuju Agricultural Research Center, Graduate School of Agriculture, Kyushu University, Taketa, Oita 878-0201 Japan; 2 Graduate School of Horticulture, Chiba University, Matsudo, Chiba 271‑8501 Japan; 3 Graduate School of Medical Life Science, Yokohama City University, Tsurumi, Yokohama, Kanagawa 230-0045 Japan; 4 RIKEN Center for Integrative Medical Sciences, Yokohama, Kanagawa 230-0045 Japan; 5 Japan Eco-science (Nikkan Kagaku) Co., Ltd, Chiba, 260-0034 Japan; 6 Sermas Co., Ltd, Chiba, 271-8501 Japan; 7 Research Center for Agricultural Information Technology, National Agriculture and Food Research Organization, Tsukuba, Ibaraki 305-0856 Japan; 8 Feed and Livestock Sector, Kanematsu Agritech Co., Ltd, Saitama, 343-0845 Japan; 9 RIKEN Center for Advanced Photonics, Wako, Saitama 351-0198 Japan; 10 RIKEN Center for Sustainable Resource Science, Yokohama, Kanagawa 230-0045 Japan

**Keywords:** Fecal bacteriome, Maternal-offspring, Octanoate, Japanese black cattle, Machine learning, Causal inference, Microbiology, Physiology

## Abstract

Maternal-offspring gut bacteriomes are closely linked to each other; however, the effects of the maternal diet have not been fully elucidated. This study aimed to determine the effect of administration of octanoate, a medium-chain fatty acid as a physiological regulator, on the fecal bacteriome and physiological indices of dams and their calves as a livestock animal model. Twelve Japanese Black cattle were randomly assigned to CON (without Ca-octanoate supplementation, *n* = 6) or OCT (with Ca-octanoate supplementation, *n* = 6), 60 days prior to the expected parturition date. A tendency towards enhanced feed efficiency was observed during the suckling period in OCT calves compared to CON calves, although the management of the calves was uniform from birth. Three types of machine learning algorithms computationally selected the feature factors of dams and their calves (30 d and 180 d of age). Based on the feature selection, causal inference estimated that feeding octanoate to dams would establish a positive association with the genus *Bacteroides*, a producer of short-chain fatty acids, at 30 d and a negative association with the genus *Candidatus Stoquefichus*, which is involved in colitis, at 180 d. These findings highlight that feeding octanoate to dams may influence the bacteriome of calves, thereby improving their feed efficiency.

**Supplementary Information:**

The online version contains supplementary material available at 10.1038/s41598-025-18490-0.

## Introduction

Gut bacteriomes play a significant role in maintaining calf health and improving feed efficiency. For example, the bacterial genera *Escherichia coli* and *Clostridium* are responsible for gut diseases by disrupting the gut barrier^1^. Conversely, the genus *Faecalibacterium* promotes an anti-inflammatory response and maintains intestinal homeostasis, thereby preventing the onset of diarrhea in calves^2^. It has been demonstrated that the family *Ruminococcaceae* and genus *Eubacterium* enhance feed efficiency through the degradation of cellulose and the production of acetic and butyric acid^3,4^. Therefore, the control of the gut bacteria involved in disease and growth during calf management is of critical importance.

Antimicrobials, probiotics, and prebiotics alter the gut microbiota and contribute to disease prevention and improved growth and feed efficiency in livestock^5–9^. Because the use of antibiotics for growth promotion has been banned in Europe owing to the risk of antibiotic-resistant bacteria^10, ^probiotics and prebiotics are attracting attention as alternatives to antibiotics. However, the effects of probiotics and prebiotics are exerted solely during the feeding period and do not persist once feeding ceases^7,11^. This phenomenon can be attributed to the stabilization of bacterial gut colonies in calves as they mature, which subsequently attenuates the efficacy of probiotics and prebiotics^12^. Therefore, continuous use of probiotics and prebiotics is essential for controlling the intestinal microbiota of calves. Conversely, regulating the gut bacteriomes of calves during stabilization could reduce the costs and labor associated with the continuous administration of biocides and non-digestible nutrients.

It is generally accepted that the gut microbiota of offspring is derived from their feeding environment, especially the symbionts of the mother^13^. The presence of bacteria orally administered to the mother from the feces of pups delivered by sterile caesarean section in mice indicates that bacterial transmission from mother to fetus may occur during pregnancy^14^. In cattle, it has been demonstrated that the feces of calves at one week of age contain bacteria from the mother^15^. Moreover, the gut bacteriome of weaned calves exhibits a strong relationship with that of the mother cow^16^. This indicated that the intestinal bacteriome of maternal cows may affect calf development. In mice, the administration of antibiotics to the mother during pregnancy has been demonstrated to results in a reduction in the diversity of bacteria and the abundance of phyla *Bacteroidetes* and *Firmicutes* within the feces of both the mother and offspring^17^. Moreover, it has been demonstrated that the administration of nine bacterial and fungal species, including the orders *Lactobacillales* and *Bifidobacteriales*, during the gestational period in maternal pigs can alter the composition of the gut microbiota of newborn piglets^18^. Therefore, it is hypothesized that the intestinal bacteriome of pups can be altered by controlling the mother’s intestinal bacteriome. Further research is required to determine the most effective methods for rearing and managing mothers, particularly, cattle.

Octanoate, a medium-chain fatty acid used as a feed additive, is typically regarded as a regulator of gut microbiota. Octanoates possess antibacterial, antifungal, antiprotozoal, and antiviral properties^19^. Administration of octanoate to fish has been shown to reduce *Escherichia*-*Shigella* and increase *Lactobacillus*^20^. In murine models, it has been demonstrated that octanoate administration results in a reduction in *Streptococcus*, a bacterial species that is a causative agent of pneumonia and septicemia, and an increase in the abundance of butyrate-producing bacteria, including *Roseburia* and *Ruminococcus*^21,22^. Additionally, octanoate administration to pigs has been shown to diminish the prevalence of *Clostridium perfringens*, a bacterium that is the primary causative agent of intestinal diseases^23^. In ruminants, an increasing number of studies are examining the effects of medium-chain fatty acids on the gut microbiota^24^. Research has demonstrated that feeding medium-chain fatty acids to Japanese Black cattle results in a reduction of pathogenic bacteria in feces^24^. In a case study focusing on octanoate as a fatty acid utilized in ghrelin synthesis^25, ^it was reported that feeding it to cattle enhanced glucose and lipid metabolism^26^. Recent reports have indicated a potential association between glucose and lipid metabolism with the composition of the gut microbiota in ruminants^27,28^. The effect of octanoate as a prebiotic is well known; however, its effect on the maternal-offspring gut bacteriome is unknown. Therefore, it may be posited that if octanoate feeding can control the gut bacteriome of maternal cows, it will result in a modified gut bacteriome in their calves, contributing to improved health status and feed efficiency.

**Fig. 1:**
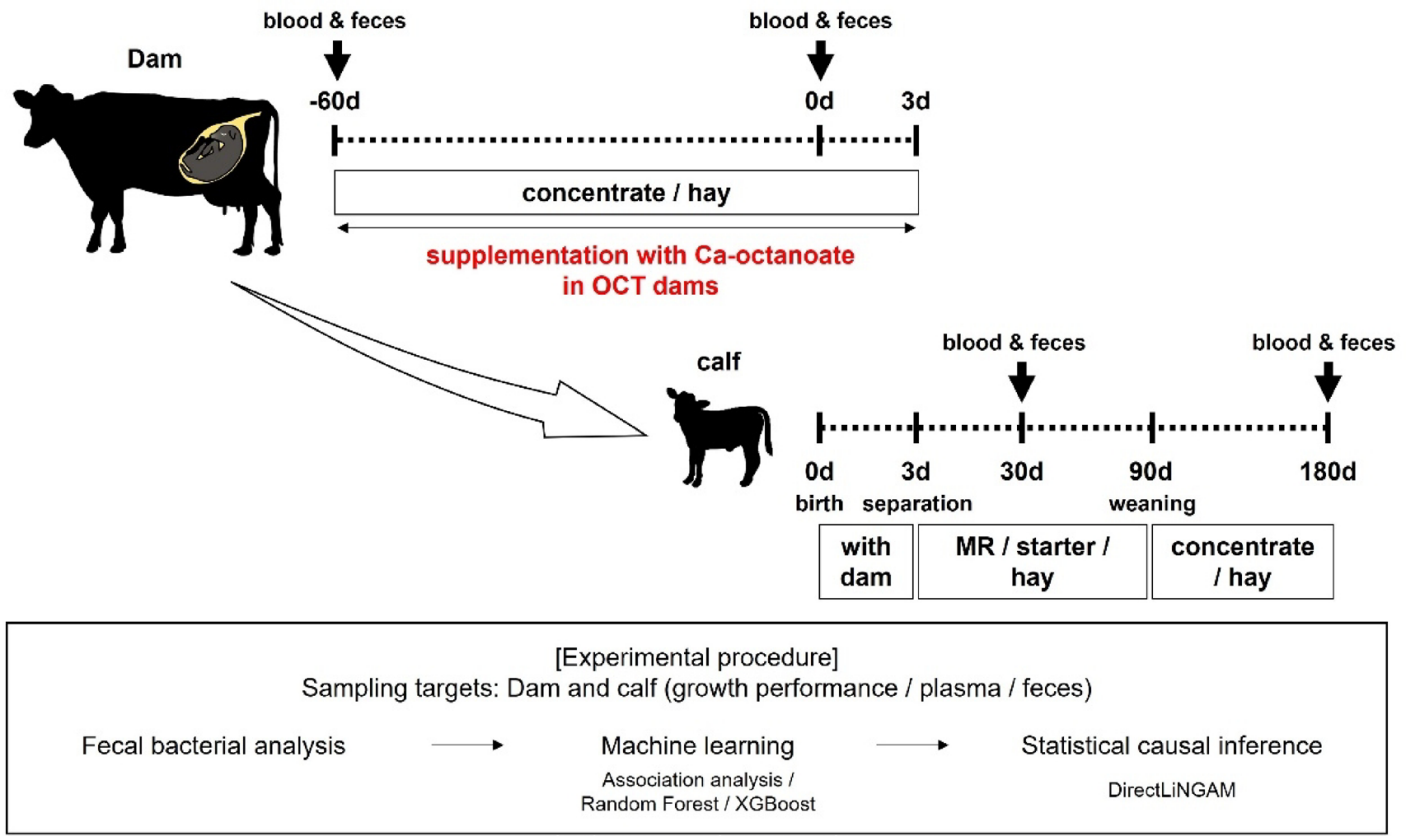
Experimental design of dams and their calves. Dams were fed concentrate without or with Ca-octanoate (CON or OCT, respectively) from 60 d prior to the expected parturition date. Calves were nursed with dams from birth to 3 d of age, fed MR, starter, and hay from 4 to 90 d of age, and fed concentrate and hay from 90 d to 180 d of age. Following the collection of fecal samples, differential analysis and three types of machine learning algorithms were conducted based on bacterial population data. Subsequently, causal inferences were made. Abbreviation indicates as follows: MR, milk replacer.

The objective of this study was to use computational techniques to assess the impact of octanoate supplementation during gestation on the development of the gut bacteriome in calves. In this study, we employed machine learning (ML) algorithms and causal inference to predict the effects of maternal octanoate supplementation on calf-rearing performance and gut bacterial composition before and after weaning. The fecal bacteriome of cows and their calves were comprehensively analyzed by 16 S RNA gene sequencing, and differential analysis/difference-in-differences (DID) was performed between calves born to dams administered octanoate (OCT in Fig. 1) and those born to dams without octanoate administration (CON in Fig. 1). The structural characteristics as a group of the fecal bacteriome of calves born from dams fed octanoate were estimated. These observations suggest that the administration of medium-chain fatty acids to maternal dams influences the composition of functional bacterial candidates in the guts of calves.

## Results

### Growth performance

Body weight and feed intake of dams have been previously demonstrated in an independent study^26^. No differences were observed in the body weight or feed intake of dams between the two treatments throughout the study.

Five male and one female calves were born to CON dams, while three male and three female calves were born to OCT dams. The body weights of OCT calves tended to be smaller than CON calves (Table 1). In contrast, when evaluated using average daily gain (ADG) without considering individual differences (e.g. foetal weight in dam) prior to octanoate administration, no statistically significant differences were observed (Table 1). Feed intake of calves during the experimental period is shown in Table 2. Hay, crude protein (CP), and crude fiber (CF) intake was lower (*P* = 0.025, 0.037, and 0.023), and starter intake and ether extract (EE) intake tended to be lower (*P* = 0.052, and 0080) in OCT calves than that in CON calves during the suckling period (from 4 to 90 days of age, Table 2). G: F (gain-to-feed ratio), an index of feed efficiency, tended to be higher (*P* = 0.098) in OCT calves than that in CON calves during the suckling period (Table 2). After weaning, hay and CF intake was lower (*P* = 0.027 and 0.026), and EE intake tended to be lower (*P* = 0.071) in OCT calves than in CON calves, but the gain-to-feed ratio did not differ between the treatments (Table 2). In addition, while CF intake was lower (*P* = 0.023) and CP and EE intake tended to be lower (*P* = 0.059 and 0.055) in the OCT calves than in CON calves, gain-to-feed ratio did not differ between treatments throughout the study period. When comparing male calves, no difference in BW was observed (see Supplementary Table S1). However, a tendency towards decreased feed intake, as well as improved feed efficiency, was observed in OCT calves (see Supplementary Table S2). These observations were consistent with the overall comparison (see Table 2). Thus, it was suggested the possibility that OCT calves enhanced feed efficiency. Based on these results, the intestinal bacteriomes of the dams and general calves were investigated.

**Table 1:** Growth performance of calves born from dams fed concentrate without (CON) or with Ca-octanoate supplementation (OCT).

Item		Treatment		*P*-value		
		CON (*n* = 6)	OCT (*n* = 6)	Treatment	Time	Treatment ×time
BW, kg	Birth	36.42 ± 1.86	33.00 ± 1.81	0.07	< 0.01	0.10
	30 d	53.29 ± 1.38	48.30 ± 2.39			
	180 d	206.62 ± 5.01	187.33 ± 8.91			
ADG, kg/d	30 d	0.56 ± 0.03	0.51 ± 0.04	0.11	< 0.01	0.57
	180 d	0.95 ± 0.03	0.86 ± 0.04			

BW, body weight; and ADG, average daily gain.

**Table 2:** Feeding performance of calves born from dams fed concentrate without (CON) or with Ca-octanoate supplementation (OCT).

Item	Treatment		*P*-value
	CON (*n* = 6)	OCT (*n* = 6)	
Preweaning period, 4–90 d			
MR intake, kg/d	0.91 ± 0.01	0.91 ± 0.00	0.59
Starter intake, kg/d	0.50 ± 0.06	0.34 ± 0.03	0.05
Hay intake, kg/d	0.47 ± 0.07	0.24 ± 0.05	0.03
CP intake, kg/d	0.38 ± 0.02	0.33 ± 0.01	0.04
EE intake, kg/d	0.18 ± 0.00	0.17 ± 0.00	0.08
CF intake, kg/d	0.20 ± 0.03	0.11 ± 0.02	0.02
G: F, kg/kg	0.49 ± 0.02	0.55 ± 0.03	0.10
Postweaning period, 91–180 d			
Concentrate intake, kg/d	3.47 ± 0.07	3.32 ± 0.18	0.45
Hay intake, kg/d	2.48 ± 0.19	1.85 ± 0.15	0.03
CP intake, kg/d	0.72 ± 0.02	0.66 ± 0.03	0.11
EE intake, kg/d	0.12 ± 0.00	0.11 ± 0.01	0.07
CF intake, kg/d	1.23 ± 0.07	0.99 ± 0.06	0.03
G: F, kg/kg	0.21 ± 0.00	0.21 ± 0.01	0.71
Total period, 4–180 d			
CP intake, kg/d	0.55 ± 0.02	0.50 ± 0.02	0.06
EE intake, kg/d	0.15 ± 0.00	0.14 ± 0.00	0.06
CF intake, kg/d	0.73 ± 0.05	0.56 ± 0.04	0.02
G: F, kg/kg	0.27 ± 0.01	0.29 ± 0.01	0.39

MR, milk replacer; CP, crude protein; EE, ether extract; CF, crude fiber; and G: F, gain-to-feed ratio (average daily gain per dry matter intake per day).

### Differential analysis

**Fig. 2:**
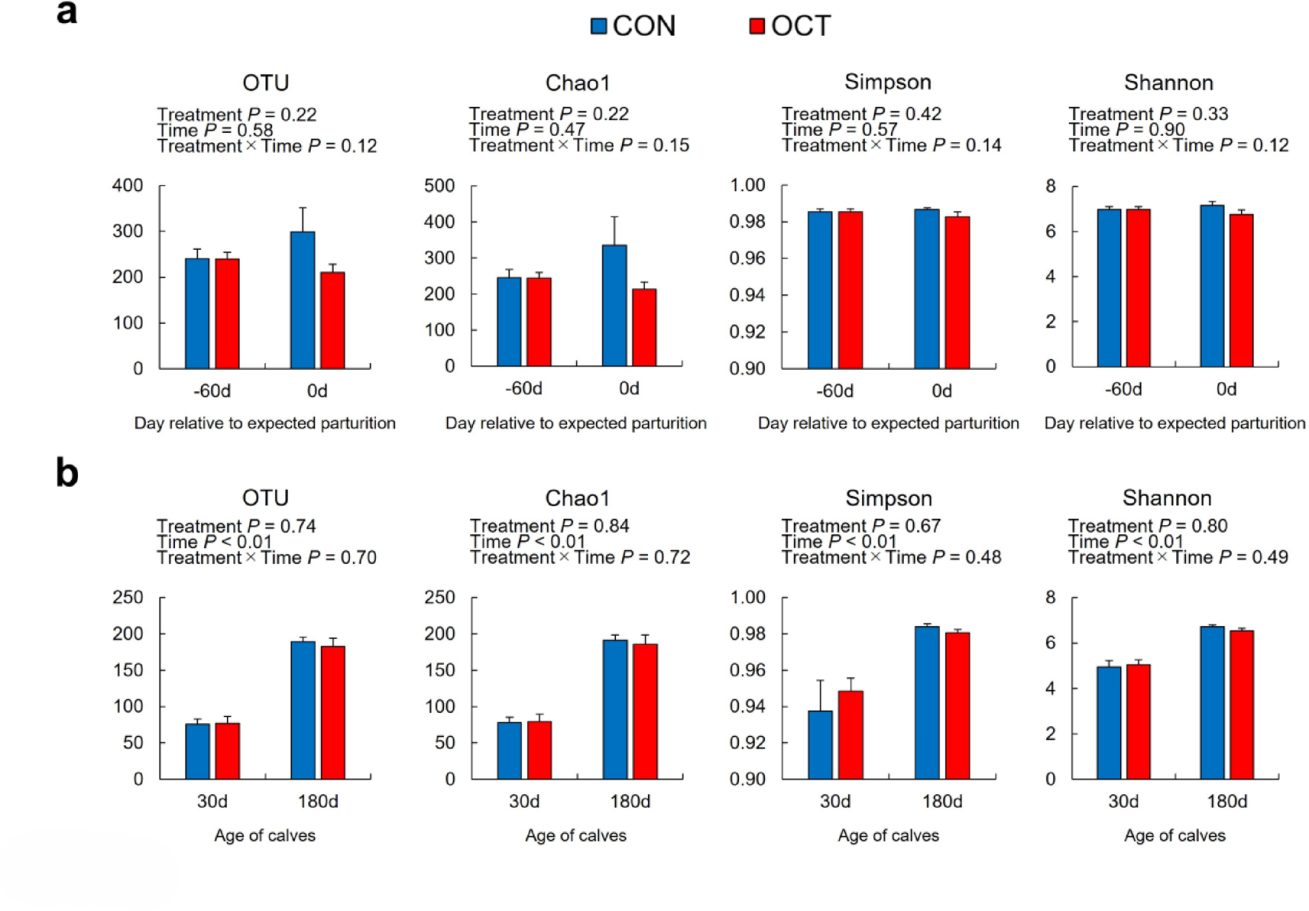
Fecal bacterial diversity in dams and their calves. Alpha diversity of (**a**) dams fed concentrate without (CON) or with Ca-octanoate supplementation (OCT) and (**b**) their calves. Data are presented as mean ± SEM.

**Fig. 3:**
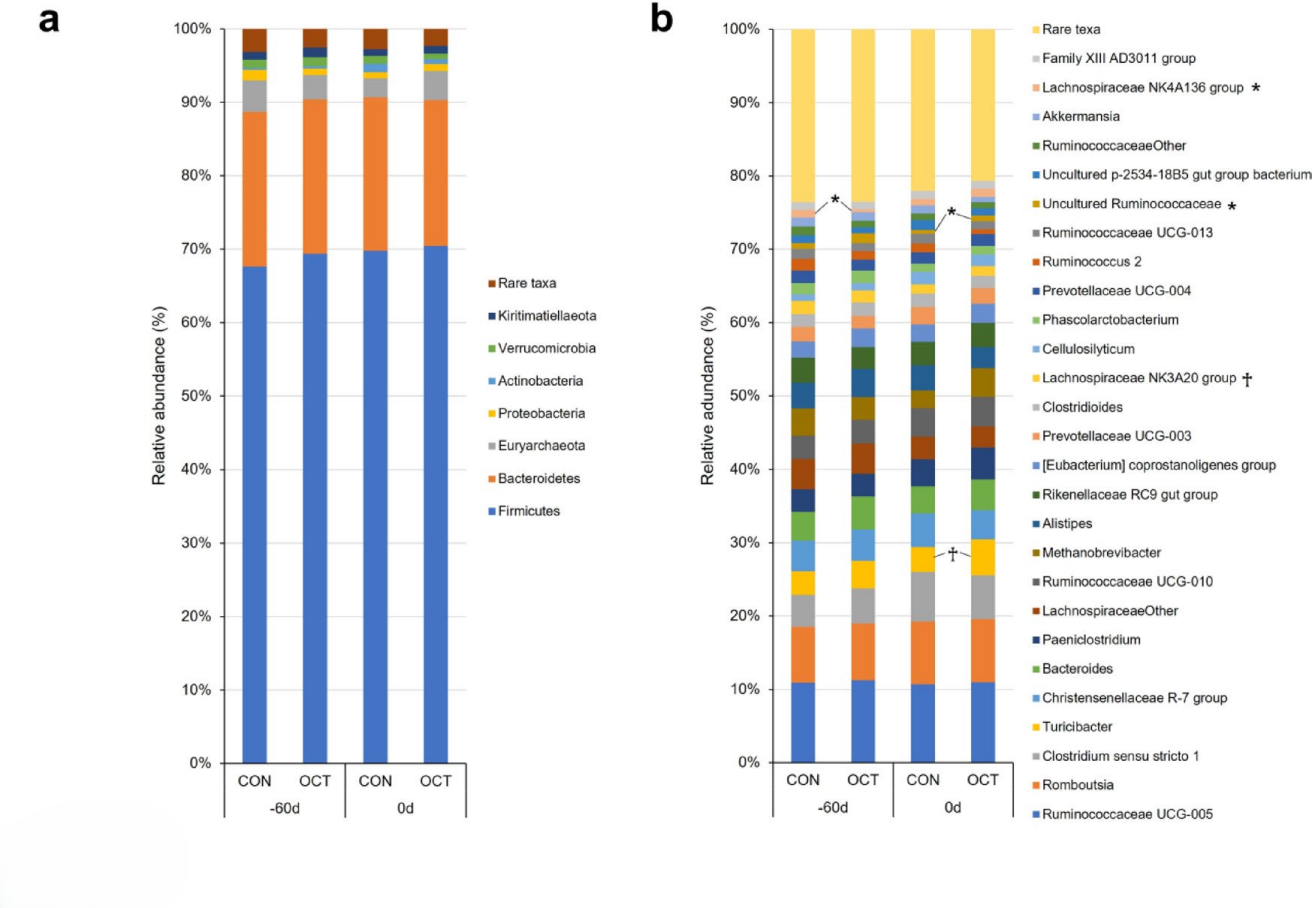
Relative abundance of dam feces. (**a**) Phyla and (**b**) genera levels in the feces of dams fed concentrate without (CON, *n* = 6) or with Ca-octanoate supplementation (OCT, *n* = 6). Asterisks (*) indicate significant differences at *P* < 0.05. The Cross (†) indicates significant difference tendencies at 0.05 ≦ *P* < 0.1.

**Fig. 4:**
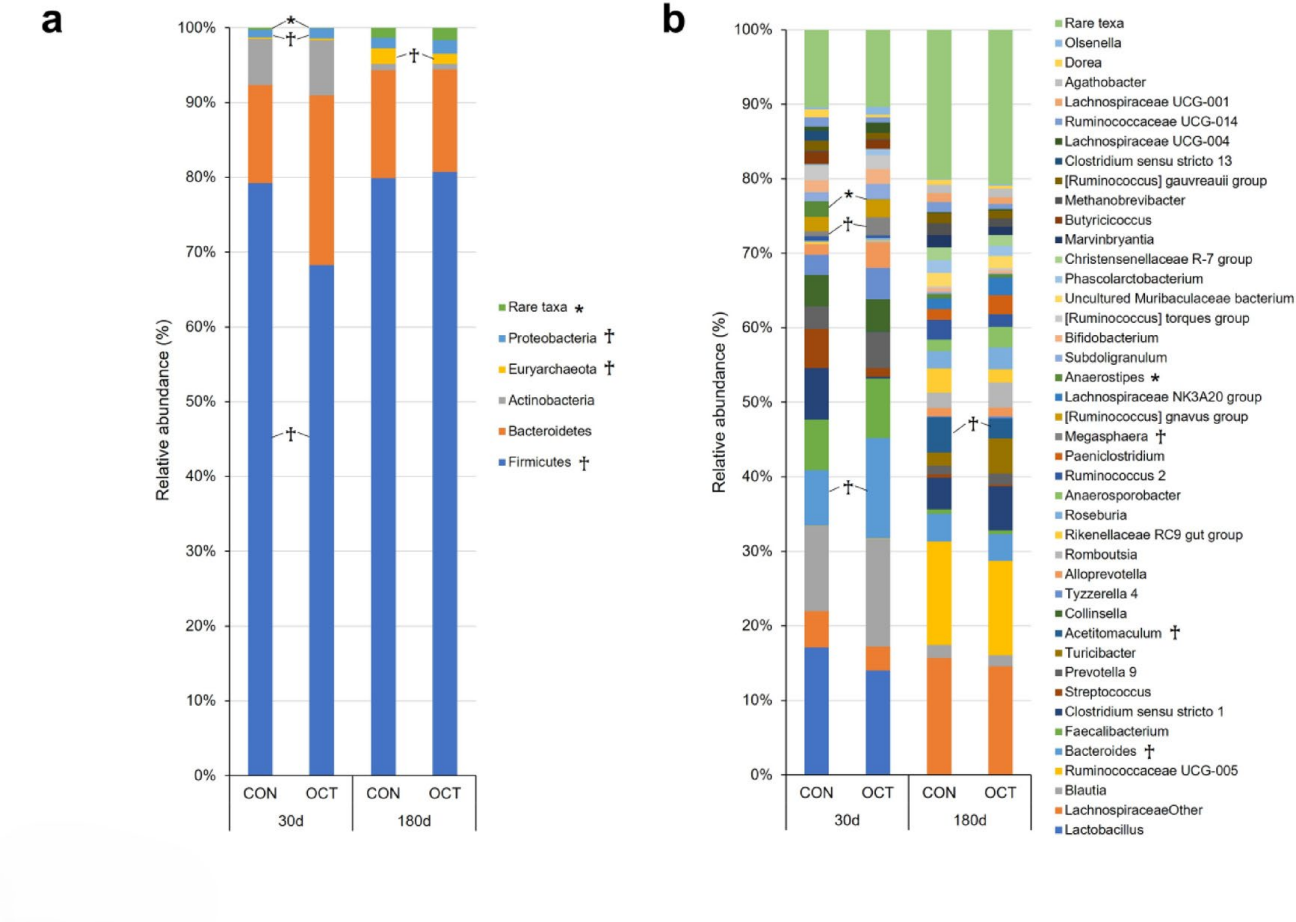
Relative abundance of calf feces. (**a**) Phyla and (**b**) genera levels in the feces of calves born from dams fed concentrate without (CON, *n* = 6) or with Ca-octanoate supplementation (OCT, *n* = 6). Asterisks (*) indicate significant differences at *P* < 0.05. Crosses (†) indicate significant difference tendencies at 0.05 ≦ *P* < 0.1.

The diversity of fecal bacteria and concentrations of organic acids in the feces of the dams remained unchanged throughout the study period (Fig. 2a and Supplementary Fig. S1). On the other hand, a decline in *Lachnospiraceae NK4A136* was observed at − 60 d in OCT dams (*P* = 0.037, Fig. 3b). An increase in *Uncultured Ruminococcaceae* at 0 d (*P* = 0.015) and a tendency towards elevated values in *Turicibacter* at 0 d (*P* = 0.078) were also observed in OCT dams (Fig. 3b). In calf feces, no significant differences were observed in the alpha diversity and concentrations of organic acids between the treatments (Fig. 2b and Supplementary Fig. S2). In contrast, the composition of bacterial phyla in the feces of OCT calves exhibited a tendency towards a decrease in the phylum *Firmicutes* at 30 days of age (30 d) and the phylum *Euryarchaeota* at 180 d, and an increase in the phylum *Proteobacteria* at 30 d relative to CON calves (Fig. 4a, *P* = 0.085, 0.090, and 0.078, respectively). At the genus level, a significant decrease in the genus *Anaerostipes* and a tendency towards an increase in the genera *Bacteroides* and *Megasphaera* were observed at 30 d in OCT calves than in CON calves (Fig. 4b, *P* = 0.031, 0.066, and 0.076, respectively). At 180 d, a tendency towards a decrease in *Acetitomaculum* in OCT calves compared to CON calves was observed (*P* = 0.055). The results indicated that bacteria, whose abundance changed significantly according to DID, were detected in the feces of both dams and calves.

### Machine learning-based bacterial classification

**Fig. 5:**
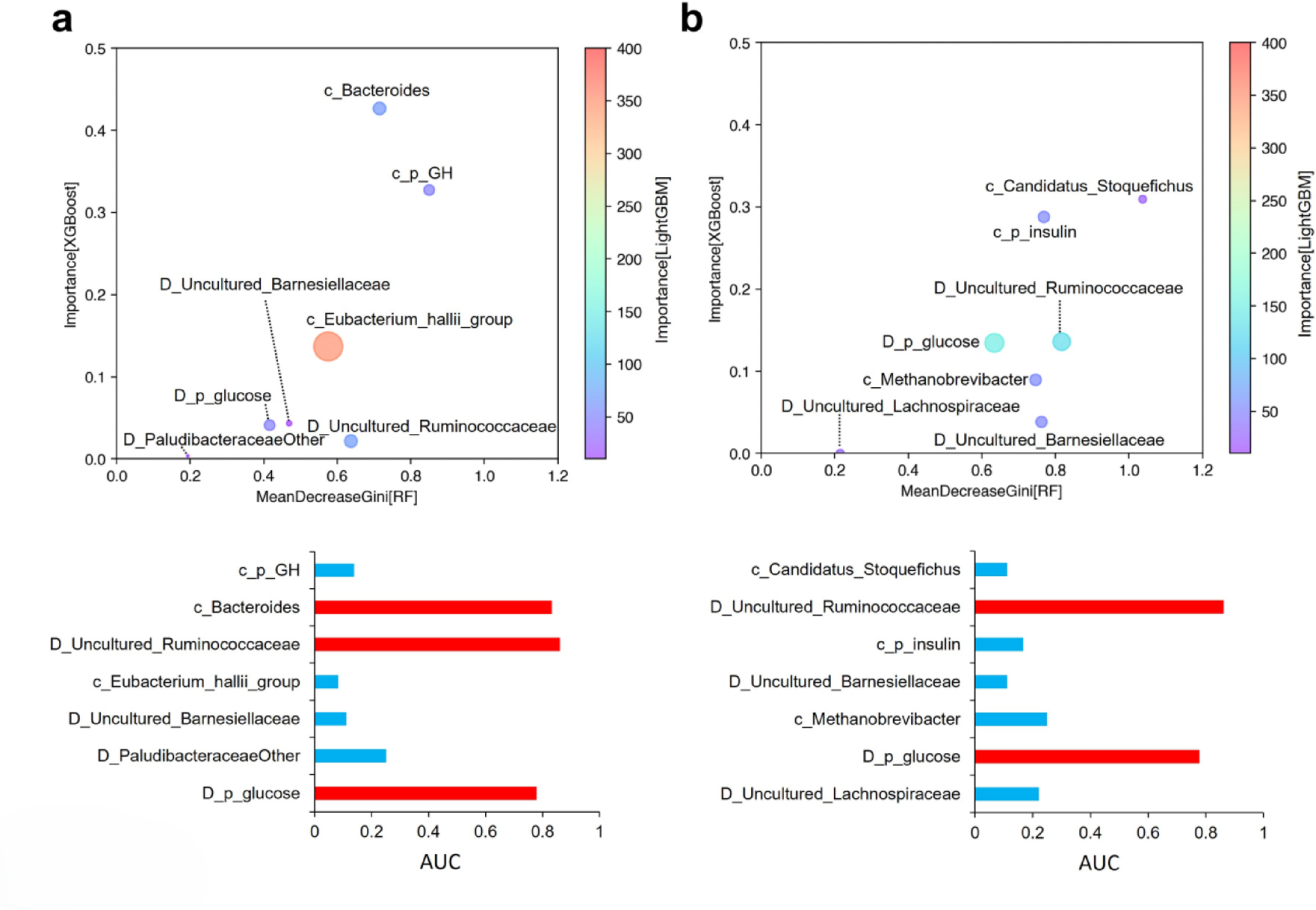
Feature selection of physical indices and bacterial genera of dams and their calves using ML algorithms. Feature selection of the fecal components and the accuracy evaluation in (**a**) 30 d calves and dam and (**b**) 180d calves and dam is visualized, respectively. The upper part shows the bubble chart of the feature component candidate values selected by ML algorithms in Fig. S5, and feature values of the components extracted by the feature selection procedure are shown on the basis of the following indicators: MeanDecreaseGini [RF], an indicator of random forest (RF); Importance [XGBoost], an indicator of extreme gradient boosting (XGBoost); and Importance [LightGBM], an indicator of light gradient boosting machine (LightGBM). The lower part shows the graph of the area under the curve (AUC) values calculated by the receiver operating characteristic (ROC) curve analysis for accuracy evaluation of the feature selection (set to CON = 0 and OCT = 1) The blue bars show components negatively associated with OCT to dam_H in Fig. S3, and the red bars show components positively associated with OCT to dam_H in Fig. S3. Abbreviations indicate as follows: D_, bacteria derived from dams; c_, bacteria derived from calves; and p_, plasma indices.

The results of DID demonstrated that only a single bacterium responded to octanoate administration in both dams and calves. In contrast, ML-based bacterial classification offers a visual representation of the relationship between the bacteriomes of dams and calves. Furthermore, ML can be used to analyze a range of data, including blood data, across a range of ages. Therefore, ML enabled a more comprehensive examination of the effects of octanoate supplementation on maternal and calf health. In this study, the network structure of the bacterial population in feces and blood components was estimated using association analysis (AA), an unsupervised ML that classifies the relationships between various groups of factors according to lift values. As described previously, AA was conducted using binarized data based on the median values for each day of age (Supplementary Fig. S3a). The results demonstrated that octanoate administration in the dam resulted in the selection of two physical indices as increasing and four bacteria as decreasing factors in the dam (Supplementary Fig. S3b). In OCT calves, two bacteria were identified as factors that increased, six indices decreased at 30 d, and three indices decreased at 180 d (Supplementary Fig. S3b). Based on these results, three types of ML algorithms, namely Random Forest (RF), XGBoost (XGB), and light gradient boosting machine (LightGBM), was used to classify and evaluate the relationship between the bacterial populations of dams and calves. On the basis of the procedure shown in Supplementary Fig. S4a, a selection of feature factors was carried out. First, the real number datasets of AA-selected common factors for “Dam&30d” and “Dam&180d” were prepared. The dataset at 30 d (“Dam&30d”) was used as training dataset, and the OOB (out-of-bag) error via RF was 33.33% (mtry = 1; number of tree = 500) and the values of “class.error” were high levels (0.5000 in CON group in 30 d and 0.1667 in OCT group in 30 d) (Table I in Supplementary Fig. S4b). Under such conditions, the dataset at 30 d (“Dam&30d”) as training dataset could accurately succeeded the classification of the dataset at 180 d (“Dam&180d”) as test dataset (Table II in Supplementary Fig. S4b). The results of these two calculations mean that the common factor of AA selection is not always accurate in distinguishing the two groups from the original training data, but it can be applied to the evaluation of the two groups beyond the age of the day. On the other hand, since AA-selected factors do not always match exactly between 30 d and 180 d, a dataset of each day age to accurately select the feature factors in detail. Thus, it can be shown below that an attempt is made to detect features using the AA-selected whole data in each age (Supplementary Fig. S4c). As a result of RF, fourteen physical indices were selected in dams and calves at 30 d (Supplementary Fig. S5a), and nine physical indices were selected for dams and calves at 180 d (Supplementary Fig. S5b). These indices were subsequently refined through the application of XGB, resulting in the identification of seven indices for dams and calves at 30 d: plasma glucose, the genera *Uncultured Barnesiellaceae*, *PaludibacteraceaeOther*, and *Uncultured Ruminococcaceae* in dams, plasma GH, the genera *Bacteroides*, and *[Eubacterium] hallii group* in calves (Supplementary Fig. S5c). Seven indices were refined by XGB in dams and calves at 180 d: plasma glucose, the genera *Uncultured Barnesiellaceae*, *Uncultured Lachnospiraceae*, and *Uncultured Ruminococcaceae* in dams; plasma insulin, the genera *Candidatus Stoquefichus*, and *Methanobrevibacter* in calves (Supplementary Fig. S5d). The feature factors selected via XGB was also confirmed via LightGBM algorithm (Fig. 5a and b, and Supplementary Figs. S5e and S5f). Furthermore, the levels of the area under the curve (AUC) for the selected factor candidates were compared via receiver operating characteristic (ROC) curves (Supplementary Figs. S5 and S5), positive factors for OCT treatment (indicated by red bars in Fig. 5a and b) exceeded 0.7, while the negative factors (indicated by blue bars in Fig. 5a and b) were below 0.3.

Thus, feature selection using multiple ML algorithm could confirm feature factor candidates, which are difficult to distinguish by DID, under the experimental conditions in this study.

### Causal inference based on the factors selected by ML

**Fig. 6:**
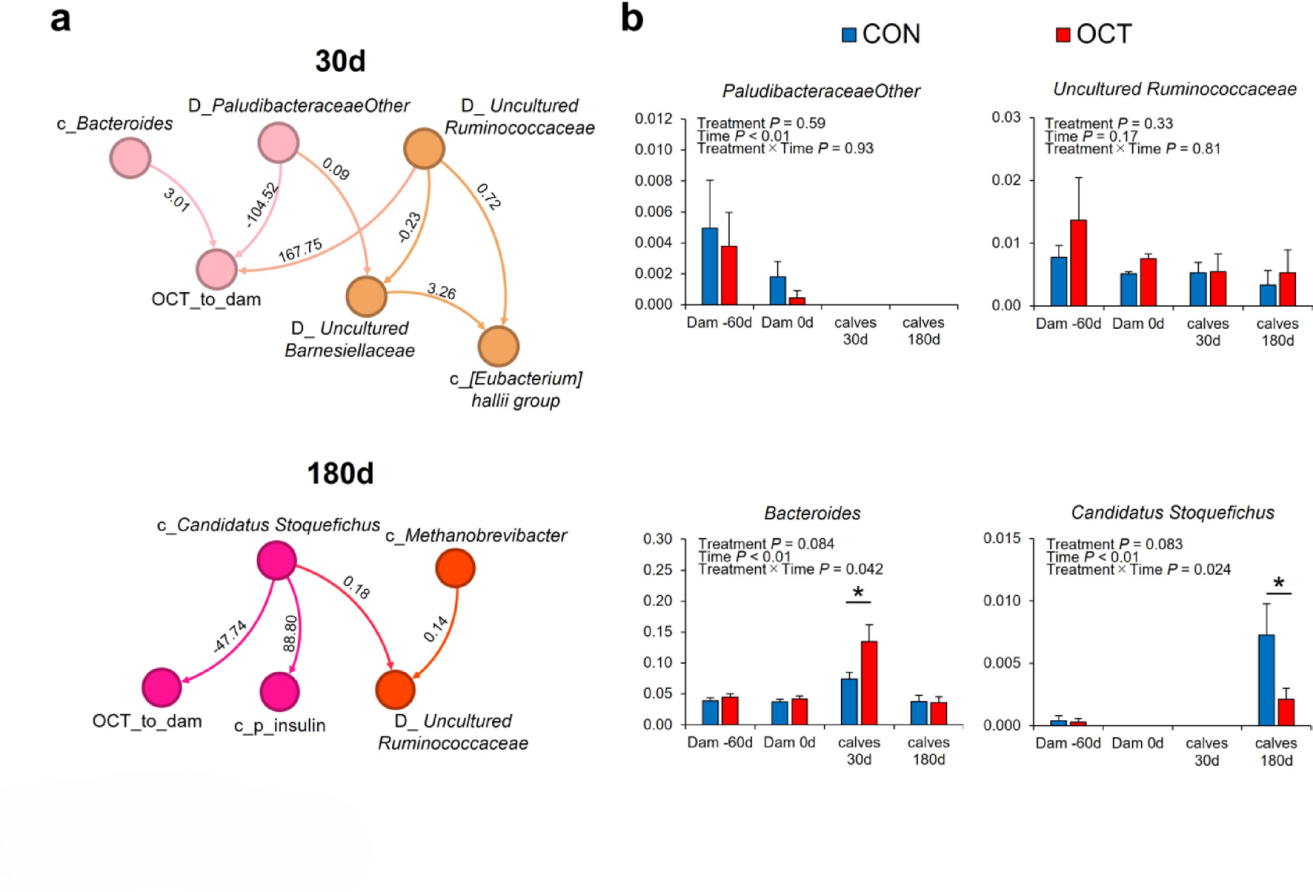
Calculated causal relationship of the components strongly linked with OCT_to_dam visualized via linear non-Gaussian acyclic model (DirectLiNGAM) analysis. (**a**) Arrow shows the causal relationship. The number shows the value of the causal contribution calculated by the DirectLiNGAM analysis. The negative and positive values show negative and positive causal contributions, respectively. Abbreviations indicate as follows: D_, the bacteria derived from the dams; c_, the bacteria derived from the calves; and p_, the plasma indices. (**b**) Relative abundance of fecal bacteria in dams fed concentrate without (CON) or with Ca-octanoate supplementation (OCT) and their calves. Data are presented as means ± SEM. Asterisks (*) indicate significant differences at *P* < 0.05.

In accordance with the aforementioned results, a relationship among the feature factors was conducted using the linear non-Gaussian acyclic model direct method (DirectLiNGAM) (Fig. 6a). DirectLiNGAM was employed to forecast the strength of the relationships between groups of factors that are not necessarily normally distributed. In general, the causal values from DirectLiNGAM mean a weak relationship for absolute values below 1 and a strong relationship for absolute values above^16,29^. In addition, the current version of DirectLiNGAM does not illustrate relationships with coefficients below 0.01^16^. DirectLiNGAM is more effective for selecting groups of strongly related factors from small datasets than for inferring precise causal relationships^29^. The results of the Shapiro-Wilk test indicated that the variables identified by XGB (Supplementary Figs. S5c and S5d) exhibited a non-normal distribution (Supplementary Table S3). Accordingly, the strength of the relationship between these factors was estimated using DirectLiNGAM for the relationship between dams and calves at 30 d and between dams and calves at 180 d. It was estimated that at 30 d, the genus *Bacteroides* in calves established a robust relationship with OCT_to_dam, namely feeding octanoate to the dam (Fig. 6a, causal value 3.01). Moreover, the genus *[Eubacterium] hallii group* in calves was associated with OCT_to_dam via *Uncultured Barnesiellaceae*, *PaludibacteraceaeOther*, and *Uncultured Ruminococcaceae* in dams. At 180 d, the genus *Candidatus Stoquefichus* in calves was found to have a strong negative relationship with OCT_to_dam (causal value, − 47.74). Furthermore, the bacterium was estimated to form a group that included plasma insulin and *Methanobrevibacter* in calves and *Uncultured Ruminococcaceae* in dams (Fig. 6a at 180 d). As shown in Fig. 6b, a repeated two-way ANOVA was conducted on the factors constituting the causal structure, as shown in Fig. 6a. The results showed that the genus *Bacteroides* at 30 d in calves was significantly increased (*P* < 0.01) and the genus *Candidatus Stoquefichus* at 180 d was reduced in OCT calves compared to CON calves (*P* < 0.01).

## Discussion

This study succeeded in comprehensively assessing the effects of oral administration of octanoate, a medium-chain fatty acid prebiotic, on the gut bacteriomes of dams and calves as a model of the maternal-offspring relationship in livestock animals. In recent years, it has been reported that the administration of antimicrobials and probiotics to the mother alters the gut microbiota of infants in mice and pigs^17,18^. Nevertheless, research examining the impact of maternal husbandry on the bacteriome of calves is limited. The present study computationally demonstrated the beneficial effects of feeding octanoate to dams on the gut bacteriomes of calves before and after weaning.

Differential analysis/differences-in-difference (DID) revealed that the oral administration of octanoate to dams resulted in a significantly higher abundance of *Uncultured Ruminococcaceae* at 0 d compared to CON dams (Fig. 3b). Members of the family *Ruminococcaceae* are cellulolytic and hemicellulolytic and produce acetic, butyric, and formic acids as well as hydrogen^30^. These bacteria have been observed to be highly prevalent in feed-efficient cattle^4^. However, the proportion of bacteria in the *Ruminococcaceae* family present in OCT calves, which exhibited a tendency towards enhanced feed efficiency, did not differ from that observed in the CON calves (Fig. 4b). *Anaerostipes*, which were reduced in calves at 30 d in OCT (Fig. 4b), were present at lower levels in feed-efficient sheep^31^. Conversely, *Anaerostipes* is a butyrate-producing bacterium^32, ^and butyrate in the gut has been shown to enhance feed efficiency, reinforce gut barrier function, and mitigate inflammation^33,34^. The fecal butyrate concentration was not consistently statistically significant (Supplementary Fig. S2), it was necessary to conduct a comprehensive evaluation of the bacteria that could influence the improvement in feed efficiency beyond the reduction in the proportion of *Anaerostipes* present in OCT calves. Consequently, ML and causal inference were employed to enhance the understanding of the impact of octanoate supplementation on the feed efficiency and gut bacteriomes of calves.

The results of this study demonstrated a robust positive correlation between the genus *Bacteroides* in calves at 30 d of age and OCT_to_dam (30d in Fig. 6a). Inabu et al. (2022) observed enhanced feed efficiency in treatments with elevated levels of *Bacteroidetes*^7^. *Bacteroides* is the most prevalent bacterium within the phylum *Bacteroidetes* (Fig. 3), and plays a role in energy production through the synthesis of propionic acid^35^. Therefore, the genus *Bacteroides* is considered to be a bacterium that improves feed efficiency in calves. The butyrate-producing *[Eubacterium] hallii group* was computationally predicted to be included in the OCT_to_dam causal structure group via bacteria from the dams (30d in Fig. 6a), but no difference was found between the two treatments in DID (Supplementary Fig. S8). Therefore, it was considered that there was no effect on butyrate production, as shown in Supplementary Fig. S2.

Conversely, *Candidatus Stoquefichus* was predicted to be a factor reduced by OCT_to_dam at 180 d of age in post-weaning calves (causal value − 47.74, 180d in Fig. 6a) and was also reduced in DID (Fig. 6b). Furthermore, a decrease in *Candidatus Stoquefichus* was strongly associated with a decrease in plasma insulin concentrations (causal value − 88.80, 180d in Fig. 6a). Changhao et al. (2024) reported that the administration of a metabolic syndrome treatment (Xiasangju) to obese rats resulted in a reduction in *Candidatus Stoquefichus* and an improved insulin resistance^36^. In summary, the decline of *Candidatus Stoquefichus* in OCT calves may have led to enhanced insulin sensitivity. This hypothesis was formulated to elucidate the potential role of this phenomenon as a contributing factor to the observed decrease in plasma insulin concentration. As previously reported^7, ^reduced plasma insulin concentrations have been observed in calves exhibiting reduced hay intake. This suggests that the observed decline in hay intake in OCT calves (Table 2) may be significantly associated with a decrease in *Candidatus Stoquefichus* and concentrations of insulin in the plasma. Furthermore, an increase in *Candidatus Stoquefichus* has recently been observed in mice with colitis, and its relationship with inflammatory bowel disease (IBD) is suspected^37,38^. Inflammatory bowel disease (IBD) is a chronic condition that leads to chronic intestinal inflammation and insulin resistance^39–41^. In addition, calves with chronic intestinal inflammation or diarrhea exhibit reduced feed efficiency^42–44^. In other words, given the reduced abundance of *Candidatus Stoquefichus* and the improved feed efficiency in OCT calves, the administration of octanoate to dams may contribute to the suppression of IBD. On the other hand, the methane-producing bacterium *Methanobrevibacter* was predicted to be included in the OCT_to_dam causal structure group via *Candidatus Stoquefichus* in calves and *Uncultured Ruminococcaceae* in dams, suggesting a potential decline in methane production (180 d in Fig. 6a). However, the effect was deemed to be minimal given that the presence ratio remained unchanged between the two treatments (Supplementary Fig. S4), and the causal value was less than 1 (180 d in Fig. 6a).

A summary of the results presented in Fig. 6 indicates that the causal value between OCT_to_dam and the bacteria present in calves at 30 d was less than that between OCT_to_dam and the bacteria present in dams. It can be reasonably deduced that the effect of octanoate supplementation on the bacterial composition of their offspring is likely to be insignificant in comparison to the effect on dams. The absence of bacteria selected as characteristic at 30 d and 180 d indicated that the effects observed at 30 d were minimal and did not persist until 180 d. In contrast, the only bacterium with a direct causal relationship with OCT_to_dam in calves at 180 d was *Candidatus Stoquefichus*. In other words, it is possible that the effects of OCT_to_dam may have become more apparent at 180 d of age. Compared with pre-weaning, post-weaning stabilizes the bacteriomes of calves^45^ and inter-individual variation disappears. In the present study, it was also found that post-weaning calves established a stronger relationship with their dams’ gut bacteria than pre-weaning calves, as reported by Taguchi et al.^16^. It was hypothesized that the administration of octanoate as a feeding management technique for dams could influence the gut bacteriome of dams and post-weaning calves.

This study is the first to demonstrate that the effect of feeding octanoate is more pronounced after weaning than before weaning. These findings suggest that the administration of octanoate to maternal cows may have resulted in the selection of bacterial strains that could potentially affect calf phenotypes. In this study, alterations in the bacterial population were of structural significance. Only one bacterium differed significantly in abundance between the two treatments on each day of age. However, using ML and causal inference, it was postulated that bacteria with low abundance ratios may interact as a group. Some of these bacteria may be transmitted by the mother’s bacteria, and some may affect the bacteria of calves without the intervention of dams. In cattle, which are more susceptible to external factors, such as rearing and the nutritional environment, there is a paucity of research on the relationship between the formation of bacteriomes in dams and calves. Given the dynamic nature of the calves’ bacteriomes, with significant changes occurring from pre-to post-weaning, continuous treatment is essential for the direct control of the intestinal bacteria of calves. Nevertheless, it is feasible to diminish the prevalence of disease and enhance growth without continuous administration of treatment to calves if the mother’s husbandry can augment the proportion of beneficial bacteria present in the calves. This study focused on medium-chain fatty acids; however, the observations are reminiscent of their use in combination with probiotics. For example, the administration of probiotic thermophile *Caldibacillus hisashii* to calves, which we have already studied, has been found to increase the relative abundance of the phylum *Bacteroidetes* and reduce the abundance of the genus *Methanobrevibacter* as methane-producing bacteria^7^. In this study, the phylum *Bacteroides* at 30 days of age was inferred to have a positive effect on the administration of octanoate, whereas the genus *Methanobrevibacter* belonged to a side categorized (via *Candidatus Stoquefichus*) that had a negative effect of the administration at 180 days of age. Therefore, it was assumed that administering medium-chain fatty acids in combination with the *Caldibacillus hisashii* to dams and/or calves could have a synergistic effect. In addition, the Picrust2 algorithm was developed to predict pathway data based on bacterial 16 S RNA gene sequence data. A comparison of the pathway data between the two treatments was conducted to assess the metabolic function of fecal bacteriomes. Volcano plot analysis revealed no statistically significant increase or decrease in metabolic pathways in dams (0 d) or calves (30 d and 180 d) (Supplementary Fig. S9; false discovery rate (FDR) > 0.1). Therefore, the effects of metabolic levels were not necessarily characterized under the experimental conditions in this study. These observations remind us of the need for other factors that affect the gut bacterial metabolism. Thus, this study computationally estimated that the administration of octanoate as a medium-chain fatty acid to dams may affect the feed efficiency of their calves, and that changes in the gut microbiota may be involved as one of the mechanisms of action, although the detailed mechanism could be not necessarily clarified. Further study would provide a new perspective to contribute to our understanding of livestock productivity, not only by investigating the effects of administering medium-chain fatty acids as a prebiotic, but also by elucidating the effects of maternal feeding management, including probiotics, on the development of the calf bacteriome.

## Methods

### Feeding management

All experimental protocols were approved by the Kyushu University Laboratory Animal Care and Use Committee (approval numbers: A-19-194-3 and A-21-042-0). The procedures used in the present study were performed according to the ARRIVE guidelines (https://arriveguidelines.org) and Guidelines for Animal Experiments by the Faculty of Agriculture at Kyushu University.

The present experiment employed a total of twenty-four Japanese Black cattle as a maternal-offspring model, i.e. six dams and six neonatal calves in the CON treatment group and six dams and six neonatal calves in the OCT group. Twelve dams were previously tested in a study conducted by Yamano et al. (2023)^26^. The twelve pregnant dams tested were selected and maintained from cattle produced at the Kuju Agricultural Research Center (Kyushu University-affiliated farm), Taketa City, Oita Prefecture, Japan (N33.055793, E131.252319). Further, twelve newborn calves from the dams were also examined during this study. The breeding of these cattle (twenty-four cattle) was maintained there on the basis of the above approved experimental plan of Kyushu University.

Twelve dams were randomly assigned to either the CON (*n* = 6) or OCT (*n* = 6) treatment group 60 days prior to calving. The cows were fed a diet comprising hay (predominantly Italian ryegrass), commercial concentrate (Yamato, JA Kitakyushu Kumiai Shiryo, Fukuoka, Japan), and soy meal (Daizu Kasu flake, Kato Oil, Osaka, Japan) once a day at 16:00, in accordance with the Japanese Feeding Standard for Beef Cattle (2008), from − 60 to 3 d after parturition (3 d). The nutritional composition of hay, concentrates, and soya meal, along with their respective proportions, has been previously described in studies conducted by Yamano et al.^26^. In OCT dams, calcium octanoate (Kanematsu Agritech Co., Ltd., Saitama, Japan) was added to the concentrate feed at a concentration of 1.5% per dry matter of the feed ration. The CON dams did not receive any additional nutrients or substances during the study period. The animals were provided water and mineral salts (Kauston, Nippon Zenyaku Kogyo, Fukushima, Japan) ad libitum.

Newborn calves were maintained with their dams for the initial three-day postpartum period and nourished with colostrum and transition milk. The calves were separated at 3 days and fed a commercial milk replacer (28.0% crude protein (CP), 18.0% crude fat (CF), and 108.0% total digestible nutrients (TDN); Calf Top EX Black, All Dairy Federation, Tokyo, Japan) from four to 90 d in accordance with the established control and octanoic acid treatments. The feeding plan for the milk replacer was based on the National Dairy Federation guidelines. Briefly, the quantity of milk replacer provided was increased from 0.5 kg/d to 1.0 kg/d between 4 and 21 d, maintained at 1.0 kg/d between 21 and 77 d, and reduced from 1.0 kg/d to 0.6 kg/d between 78 and 90 d. The calves were fed a commercial starter (18.0% CP, 2.0% CF, 74.0% TDN; Shinn-jinkounyu Koushinomachi, JA Kitakyushu Kumiai Shiryo, Fukuoka, Japan) and Italian ryegrass hay (65.8% dry matter (DM), 10.2% CP, 2.3% CF, 63% TDN per DM) ad libitum from 4 to 90 d. From 91 to 180 d, the calves were fed up to 4 kg/day of commercial concentrate (Magokoro, JA Kitakyushu Kumiai Feed, Fukuoka, Japan; 16.0% CP, 2.5% CF, and 68.0% TDN) ad libitum on Italian ryegrass hay. The calves were fed twice a day (9:00 and 16:00) and had free access to water and minerals.

### Sample collection

The cows were weighed weekly before calving and on the day of calving (0d). Feces of dams were collected at − 60 d and 0 d. Feces were collected using a fecal collection tube with disposable gloves and a spoon (SARSTEDT AG & Co. KG, Germany). The collected feces were stored at − 30 °C until analysis.

The weight of the calves was recorded at 0, 30, and 180 d. Blood and fecal samples were obtained before the morning feeding period at 30 and 180 d. Blood samples were collected via the jugular vein using a 21 G blood collection needle (MN-2138MF, Terumo, Tokyo, Japan) and a 10 mL vacuum blood collection tube (Venoject II VP-H100K with heparin sodium; Terumo, Tokyo, Japan). The blood samples were immediately placed on ice. Blood was transferred to a centrifuge tube (AGC TECHNO GLASS Co., Ltd., Shizuoka, Japan) supplemented with heparin sodium (10 IU/mL of blood; AY Pharmaceuticals Co., Ltd., Tokyo, Japan) and aprotinin (100 Kallikrein Inhibitor Unit/mL of blood; Wako Pure Chemical, Osaka, Japan) and centrifuged at 4 ℃ and 2330×g for 20 min. The plasma was stored at − 80 ℃. Feces of calves were collected immediately following the collection of blood samples using rubber gloves and a fecal collection tube. The feces were stored at − 30 ℃ until analysis.

### Hormones and metabolites in plasma assay

The concentrations of growth hormone (GH) and insulin in plasma were analyzed by time-resolved fluorescence immunoassay (TR-FIA) according to previous studies^26,46^. The inter- and intra-assay coefficients of variation for GH were 8.6% and 6.5%, respectively, while those for insulin were 9.3% and 7.7%, respectively. Plasma glucose concentrations were measured using a commercial assay kit (Glucose C2 test Wako, Wako Pure Chemical, Osaka, Japan).

### Organic acid in feces

Fecal samples (200–400 mg) were analyzed according to previous reports^7,47^. Samples were mixed with 9x Milli-Q water for 10 min, centrifuged, and filtered. The filtered solution was analyzed for lactic, acetic, propionic, butyric, valeric, and isovaleric acids using high-performance liquid chromatography (HPLC). Analyses were conducted using an HPLC Prominence instrument (Organic Acid Analyzer; Shimadzu, Kyoto, Japan) with an ion-exclusion column (Shim-pack SCR-102 H; Shimadzu) at 40 ℃ and an electrical conductivity detector (CDD-10AVP; Shimadzu) in conjunction with an organic acid analyzer at a flow rate of 0.8 ml/min. The mobile phase was 5 mM *p*-toluenesulfonic acid, and the buffer was 5 mM *p*-toluenesulfonic acid, 20 mM Bis-Tris, and 0.2 mM EDTA-4 H. and a flow rate of.

### Analysis of fecal bacterium

DNA was extracted from fecal samples using the QIAamp PowerFecal DNA Kit (QIAGEN N.V., Inc., Hilden, Germany), according to the manufacturer’s protocol. DNA concentrations were evaluated using the Quant-iTTM PicoGreen dsDNA Assay Kit (Thermo Fisher Scientific, Tokyo, Japan). The nucleotide sequence of the V3-V4 region (314F-806R) of the bacterial 16S rRNA gene was determined as previously described^48^. The obtained sequences were analyzed via Qiime 1.9.1 (https://twbattaglia.gitbooks.io/introduction-to-qiime/content/). In short, the feature classifier was run after checking or denoising the sequence data with DADA2. The classifier was executed using SILVA_132 as the reference genome database (SILVA_132) under the default condition. The filtering of 10 total counts or less (p-min-frequency 10) and the elimination of mitochondria and chloroplasts (p-exclude mitochondria, chloroplast) were also performed. Subsequent analyses were then performed on the obtained data files. Alpha diversity (α-diversity) and bacterial communities were analyzed by the library “easyGgplot2”, “knitr”, “ggthemes”, “phyloseq”, “genefilter”, “gplots”, “ggplot2”, “RColorBrewer”, “pheatmap”, “ape”, “base”, “dplyr”, and “vegan” of the R software (version 4.2.2) (https://cran.r-project.org). The observed operational taxonomic units (OTUs) and Chao1, Shannon, and Simpson indices were used as α-diversity indices. Statistical analysis of these values was performed using the “adonis” function of the library “vegan” library in the R software package. The relative abundances of dominant and/or characteristic bacteria (> 1% of the detected community for relative abundance analysis) were analyzed using the R software (version 4.4.0). Fecal bacterial genus composition data were clustered using ‘gplots’ and ‘genefilter’ libraries based on correlation coefficients. Metabolic pathway prediction was performed using the PICRUSt2 algorithm^49^. Analysis was performed using the library packages “limma” and “edgeR” in the R software. Volcano plots were generated using the “tidyverse” and “ggplot2”.

The 16 S rRNA gene dataset was deposited in the DNA Data Bank of Japan (DDBJ) under NCBI BioProject accession number PRJDB19664 (BioSample accession number: SAMD00849944-SAMD00850051).

### ML algorithms

ML algorithms were applied in accordance with previous reports^16,29,50–52^.

Association analysis is an established method in predictive science. It is typically employed to elucidate the relationships between components by using relative numbers^16,53,54^. Association rules were determined using the values of support, confidence, and lift. The analysis was conducted using R software (version 4.3.2) with the packages ‘arules’ and ‘aruleViz’. All data were calculated based on the median (M) for the same-day age category and were subsequently sorted as 0 (≤ M) and 1 (> M). The relevant analysis parameters were set as support = 0.1, confidence = 0.8; and lift, > 1.2. The entire network was visualized using the “Force Atlas” and “Noverlap” plugins of Gephi 0.10.1 (https://gephi.org/).

Random forest is an algorithm that combines multiple decision trees and bagging methods^55^. The R software package ‘randomForest’ was used in this study. The significance of individual variables was evaluated based on the mean value of the Gini reduction. The mean value of the Gini reduction serves as an indicator of the importance (ability) of a variable in partitioning data within the model. A higher mean value of the Gini reduction indicates greater importance of the variable in the data partition^56^. XGBoost, eXtreme gradient boosting, is an algorithm that combines boosting with a set of decision trees^57^ and was computed using the ‘xgboost’ package, which is an optimized distributed gradient boosting library designed to be highly efficient, in R software^58^. In addition, light gradient boosting machine (LightGBM) for a highly efficient gradient boosting decision tree^59, ^were also used. The following libraries and modules were used via Python (version 3.10.8) in Mac OS Sequoia (version 15.3) on arm64: “lightgbm”, “train_test_split”, “accuracy_score”, “pyplot”, “pandas”, “yaml”, and “csv”. These selected feature factors were visualized as a bubble chart using the “matplotlib” (https://matplotlib.org/stable/index.html).

To evaluate the diagnostic accuracy of the feature factors, receiver operating characteristic (ROC) curve analysis was performed via the R library “Epi”. The calculated area under the curve (AUC) was used to evaluate the effect of OCT treatment.

### Causal inference for the ML-selected bacteriomes

To estimate structural models that extend beyond the limited distribution of experimental data^52,54, ^the DirectLiNGAM (a direct method for learning the basic LiNGAM model) approach for linear non-Gaussian acyclic model learning^16,29,53^ employs independent component analysis and non-Gaussian methods to estimate causal structures. In this study, the relationships between factors across days of age were assessed using the properties of DirectLiNGAM applied to independent components. DirectLiNGAM (https://github.com/cdt15/lingam) was built in Python (version 3.10.8), using Mac OS Sequoia (version 15.3). The data calculated by the DirectLiNGAM analysis were used in the Python library numpy (version 1.26.4), pandas (version 2.2.3), matplotlib (version 3.9.2), cikit-learn (version 1.6.1), lingam (version 1.8.0), and “graphviz (version 0.19.1)” to ensure the validity of the acyclic graphs (DAGs). Subsequently, the directed acyclic graph datasets for the DAGs were visualized using the Gephi software (version 0.10.1) (https://gephi.org).

### Statistical analysis

Data on body weight and concentrations of plasma components and fecal organic acids were analyzed using JMP^®^14 Pro (SAS Institute Inc., NC, USA) according to the following model (Eq. 1):

1 $${\text{Y}}_{{{\text{ijk}}}} = {\text{ }}\mu {\text{ }} + {\text{ Treatment}}_{{\text{i}}} + {\text{ Time}}_{{\text{j}}} + {\text{ Calf }}\left( {{\text{Treatment}}} \right)_{{{\text{k}}({\text{i}})}} + {\text{ Treatment }} \times {\text{ Time}}_{{{\text{ij}}}} + {\text{ e}}_{{{\text{ijk}}}}$$

where Y_ijk_ is the dependent variable, µ is the overall mean, Treatment_i_ is the fixed treatment effect, Time_j_ is the fixed effect of time used as a repeated measure, Calf(Treatment)_k(i)_ is the variable effect of individuals branched by treatment, Treatment × Time_ij_ is the interaction between treatment and time, and e_ijk_ signifies the error. Simple main effect tests were performed to identify differences between treatments at the same time as an interaction.

The relative abundances of dominant and/or characteristic bacteria (> 1% of the detected community for relative abundance analysis) were analyzed using the R software (version 4.4.0). The Gaussian distribution of abundance was evaluated using the Shapiro–Wilk test to select parametric and nonparametric analyses. Student’s t-test or Welch’s t-test was used as a parametric test following the evaluation of equal variances. The Wilcoxon rank-sum test was used for nonparametric tests. Significance and tendencies were declared at *P* < 0.05 and 0.05 ≤ *P* < 0.10, respectively. All data are presented as the mean ± standard error of the mean.

## Supplementary Information

Below is the link to the electronic supplementary material.

Supplementary Material 1.

Supplementary Material 2.

Supplementary Material 3.

## Data Availability

Raw files of the bacterial V3–V4 16 S rRNA sequences were deposited in the DNA Data Bank of Japan (DDBJ) under NCBI BioProject accession number PRJDB19664 (BioSample accession number: SAMD00849944-SAMD00850051). The datasets generated and analysed during this study are stored in a supplemental data file and are available from the corresponding author upon reasonable request.
