# Supplementary material for: Maternal administration of octanoate, a medium-chain fatty acid, improves feed efficiency of Japanese black calves through influencing gut bacteriome structure
Source: Sci Rep. 2025 Sep 29;15:33557. doi: 10.1038/s41598-025-18490-0 (PMC12480703; doi:10.1038/s41598-025-18490-0)
Supplement: Supplementary file 1 — Supplementary Material 1. [file 41598_2025_18490_MOESM1_ESM.pdf]

Supplementary Information

Maternal administration of octanoate, a medium-chain fatty acid, improves feed efficiency of Japanese Black calves through influencing gut bacteriome structure

Haruki Yamano[1], *Hiroshi Horike*[1], *Yutaka Taguchi*[1], *Yudai Inabu*[1], *Hirokuni Miyamoto*[2,3,4,5,6*], *Atsushi Kurotani*[7], *Nonomi Suzuki*[8], *Shigeharu Moriya*[9], *Teruno Nakaguma*[2,5,6], *Chitose Ishii*[4,6], *Makiko Matsuura*[2,6], *Naoko Tsuji*[6], *Tetsuji Etoh*[1], *Yuji Shiotsuka*[1], *Ryoichi Fujino*[1], *Satoshi Wada*[9], *Jun Kikuchi*[3,10], *Hiroshi Ohno*[4], and *Hideyuki Takahashi*[1*]

[1]*Kuju Agricultural Research Center, Graduate School of Agriculture, Kyushu University, Taketa, Oita 878-0201, Japan*
[2]*Graduate School of Horticulture, Chiba University, Matsudo, Chiba 271-8501, Japan*
[3]*Graduate School of Medical Life Science, Yokohama City University, Tsurumi, Yokohama, Kanagawa 230-0045, Japan*
[4]*RIKEN Center for Integrative Medical Sciences, Yokohama, Kanagawa 230-0045, Japan*
[5]*Japan Eco-science (Nikkan Kagaku) Co., Ltd., Chiba 260-0034, Japan*
[6]*Sermas Co., Ltd., Chiba 271-8501, Japan*
[7]*Research Center for Agricultural Information Technology, National Agriculture and Food Research Organization, Tsukuba, Ibaraki, 305-0856, Japan*
[8] *Feed and Livestock Sector, Kanematsu Agritech Co., Ltd., Saitama, 343-0845, Japan*
[9]*RIKEN Center for Advanced Photonics, Wako, Saitama 351-0198, Japan*
[10]*RIKEN Center for Sustainable Resource Science, Yokohama, Kanagawa 230-0045, Japan*

*Co-corresponding author:
Hirokuni Miyamoto, Graduate School of Horticulture, Chiba University, Matsudo, Chiba 271-8501, Japan.
Email: h-miyamoto@faculty.chiba-u.jp and hirokuni.miyamoto@riken.jp
Hideyuki Takahashi, Kuju Agricultural Research Center, Graduate School of Agriculture, Kyushu University, Taketa, Oita 878-0201, Japan.
Email: takahashi.hideyuki.990@m.kyushu-u.ac.jp

Contents

Fig. S1.
Concentrations of fecal organic acids in dams.

Fig. S2.
Concentrations of fecal organic acids in calves.

Fig. S3.
Associated networks of physical indices sorted with octanoate supplementation to dams (OCT to dam_H).

Fig. S4.
Screening of feature factors using Random Forest (RF), XGBoost (XGB), and light gradient boosting machine (LightGBM).

Fig. S5.
Feature importance ranking of physical indices and bacterial genera of dams and their calves using the Random Forest (RF), XGBoost (XGB), and light gradient boosting machine (LightGBM) methods.

Fig. S6.
Receiver operating characteristic (ROC) curve analysis for ML-selected feature factors in Fig. 5a.

Fig. S7.
Receiver operating characteristic (ROC) curve analysis for ML-selected feature factors in Fig. 5b.

Fig. S8.
Relative abundance of the fecal bacterial genera indirectly linked with "OCT_to_dam" in Fig. 6a.

Fig. S9.
Volcano plot of pathways for dams and their calves.

Table S1.
Growth performance of male and female calves born from dams fed concentrate without (CON) or with Ca-octanoate supplementation (OCT).

Table S2.
Feeding performance of male and female calves born from dams fed concentrate without (CON) or with Ca-octanoate supplementation (OCT).

Table S3.
Statistical evaluation of the distribution of fecal bacteria and plasma indices data from the dams and their calves.

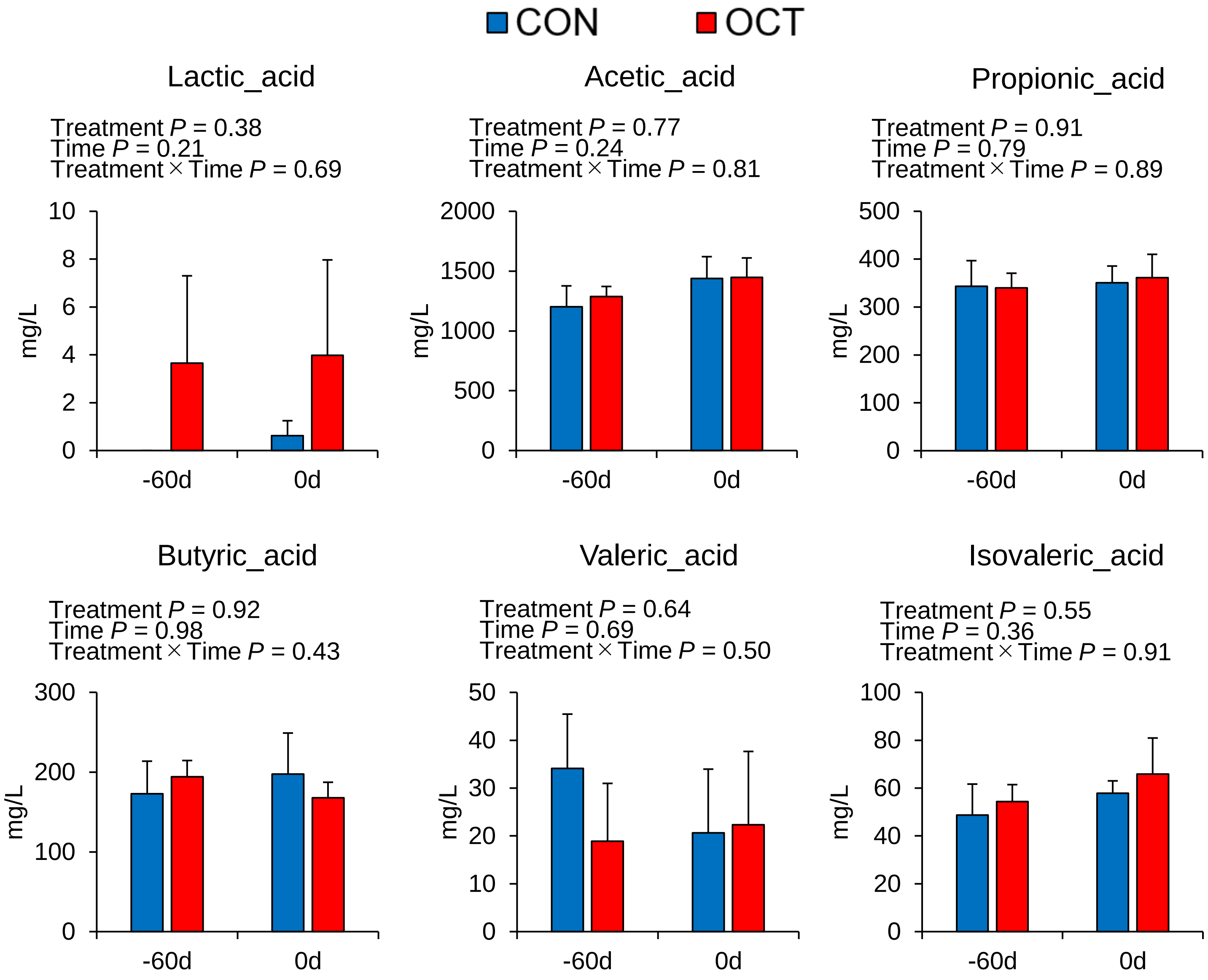

Fig. S1.

Concentrations of fecal organic acids in dams.

The dams were fed concentrate without (CON) or with Ca-octanoate supplementation (OCT). Data are presented as means ± SEM.

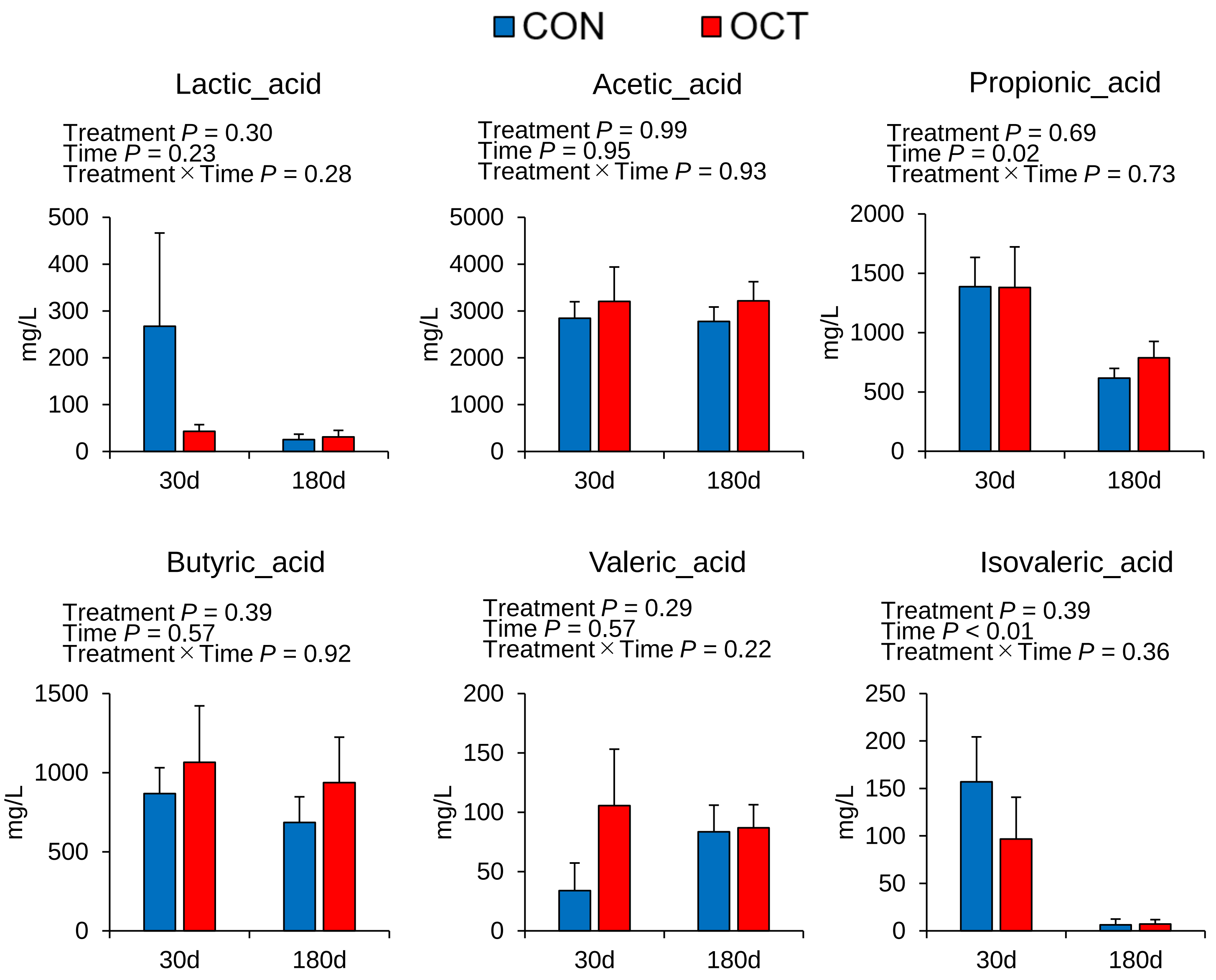

Fig. S2.

Concentrations of fecal organic acids in calves.

The calves were born to dams fed concentrate without (CON) or with Ca-octanoate supplementation (OCT). Data are presented as means ± SEM.

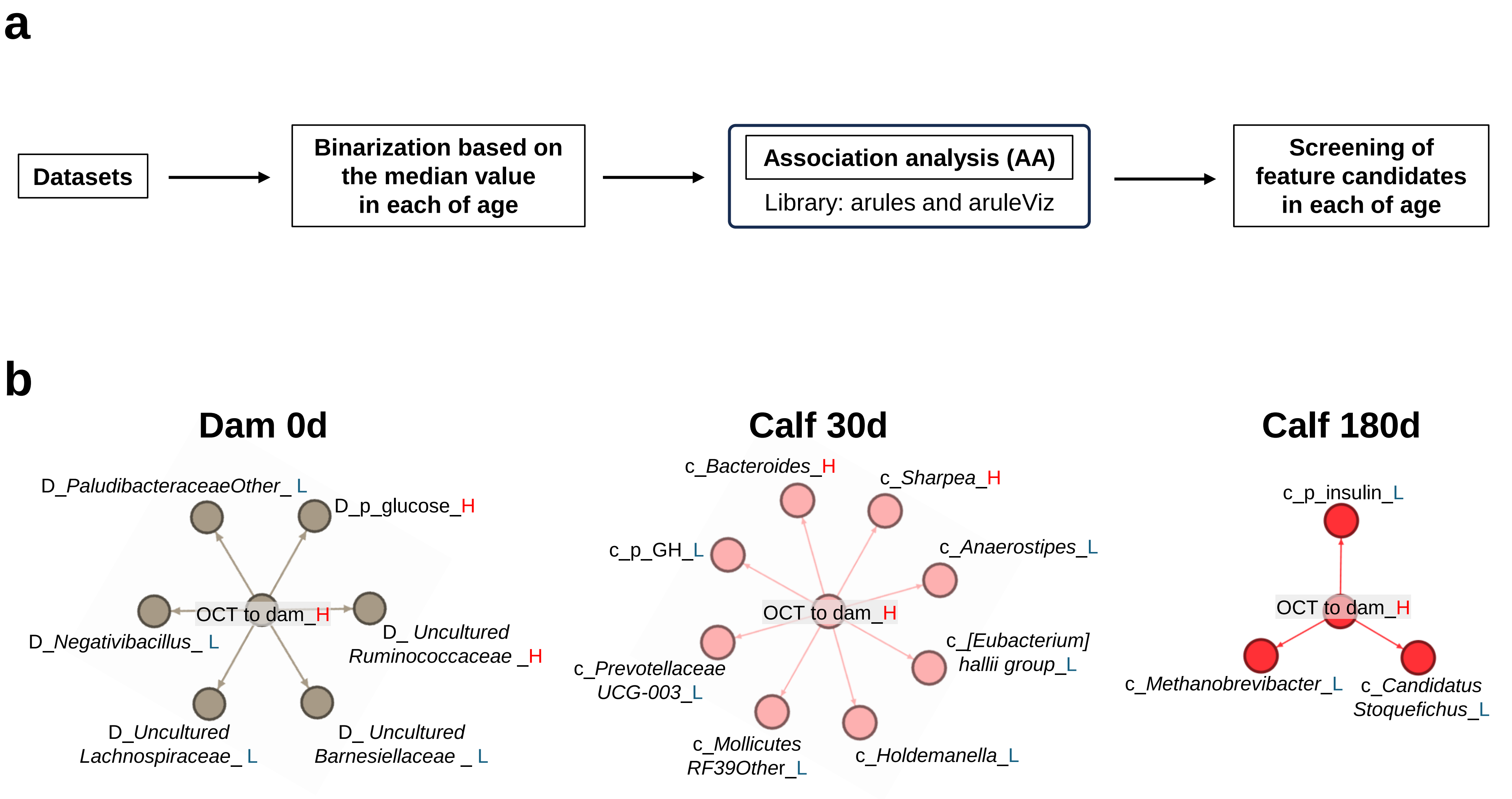

Fig. S3.

Associated networks of physical indices sorted with octanoate supplementation to dams (OCT to dam_H).

(a) Workflow for selection of feature candidates via association analysis. (b) In the association analysis, physical indices were sorted according to the lift value (> 1.2). Positive and negative associated relationships are divided into high (H) (red) or low (L) (blue) levels based on the mediation values of the entire dataset of targeted components. The abbreviations are as follows: D_, bacteria derived from dams; c_, bacteria derived from calves; p_, plasma indices; GH, growth hormone.

a

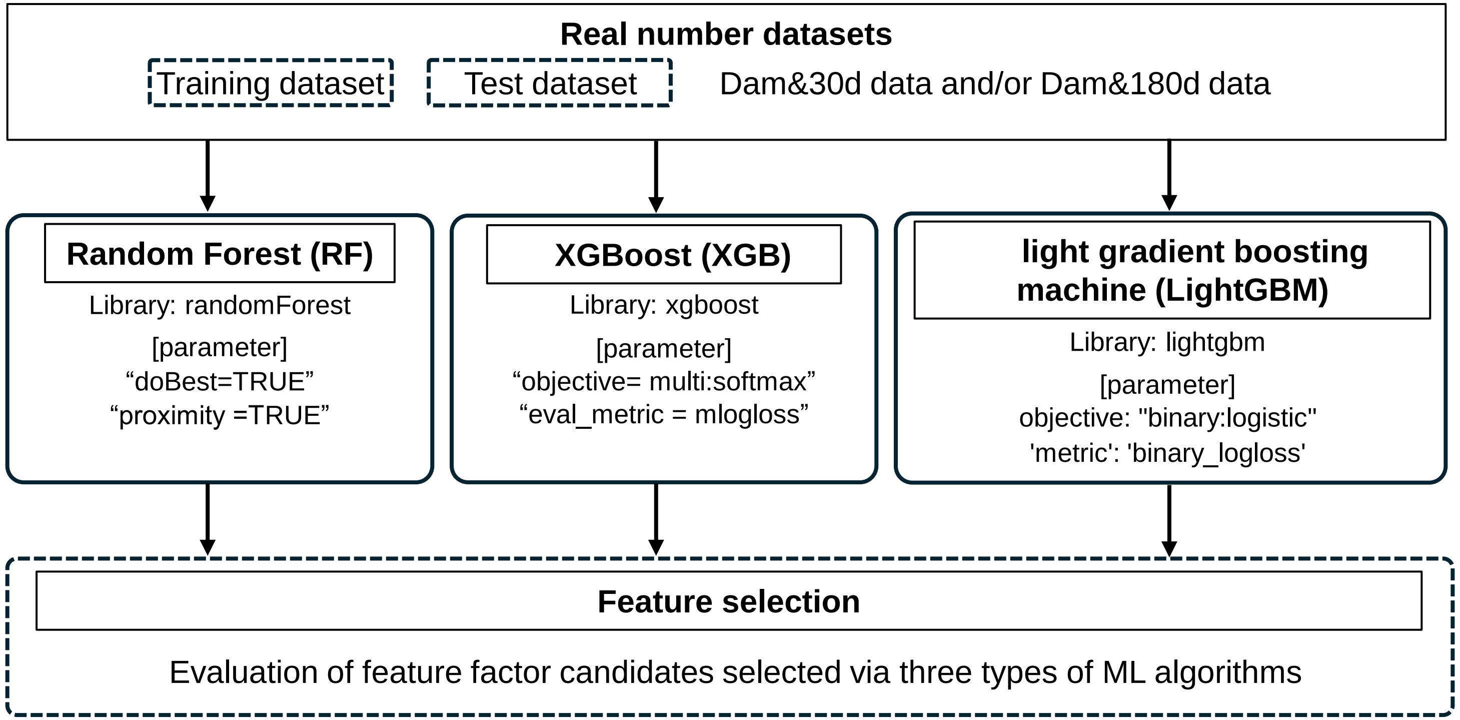

b

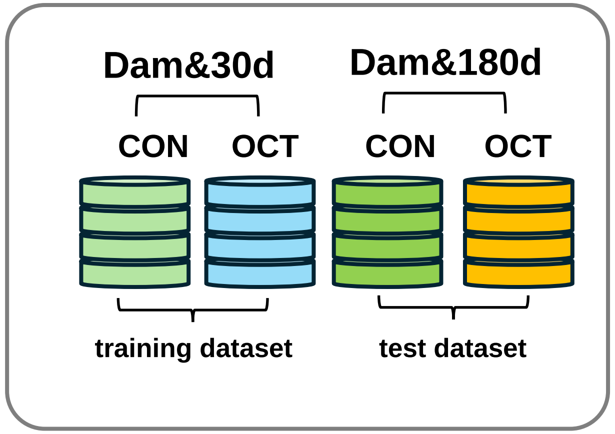

Table I

tuneRF	30d_CON	30d_OCT	class.error
30d_CON	3	3	0.5000
30d_OCT	1	5	0.1667

Table II

prediction	30d_CON	30d_OCT	class.error
180d_CON	6	0	0
180d_OCT	0	6	0

c

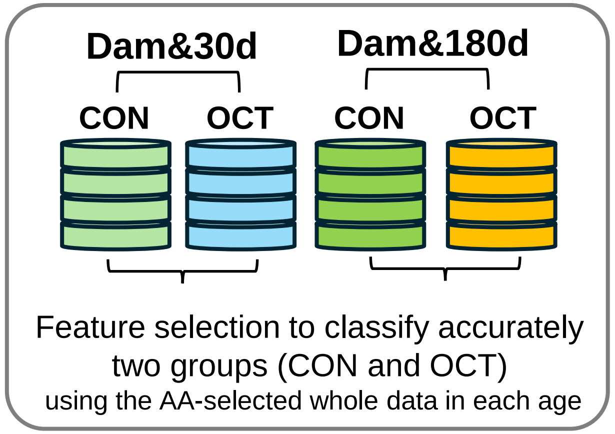

Fig. S4.

Screening of feature factors using Random Forest (RF), XGBoost (XGB), and light gradient boosting machine (LightGBM).

(a) Workflow for feature selection using RF, XGB, and lightGBM as ML algorithms. An overview of the analytical steps and paremeter conditions was visualized. (b) Treatment of training dataset and test dataset. Here, the real number datasets of the AA-selected common factors for “Dam&30d” and “Dam&180d” were prepared. Table I shows the confusion matrix of training dataset [“Dam&30d”] based on the function ‘tuneRF’ (parameter: doBest) of Random Forest. The “class.error” in the table indicates the error rate in each class of training dataset. Table II shows the predicted confusion matrix for the test dataset [“Dam&180d”] based on the training dataset [“Dam&30d”]. (c) The adjustment of the training dataset and the test dataset to classify two groups within Dam&30d and Dam&180d. The abbreviations are as follows: training dataset, a dataset for training via the MLs for a model; test dataset, a dataset for testing via the MLs to be predicted; Dam&30d, dams in 0d and calves in 30d; Dam&180d, dams in 0d and calves in 180d; CON, the data of a CON group; OCT, the data of an OCT group; 30d_CON, the data of CON group of Dam&30d; 30d_OCT, the data of OCT group of Dam&30d; 180d_CON, the data of CON group of Dam&180d; 180d_OCT, the data of OCT group of Dam&180d; and “class.error,” an error rate in each class in the table.

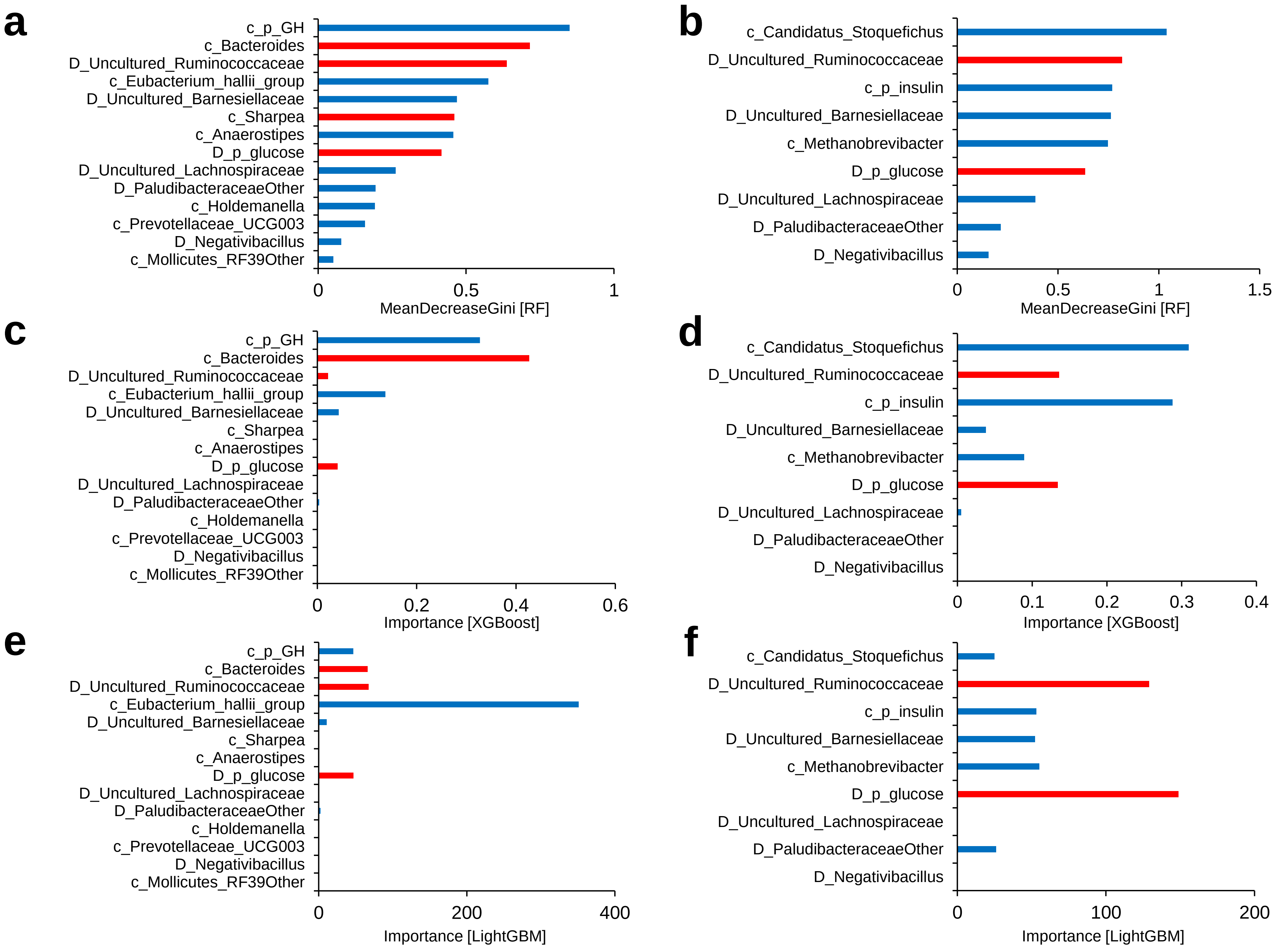

Fig. S5.
Feature importance ranking of physical indices and bacterial genera of dams and their calves using the Random Forest (RF), XGBoost (XGB), and light gradient boosting machine (LightGBM) methods.
The relative importance of the RF feature is demonstrated in (a) [30d and dam] and (b) [180d and dam], whereas an analogous demonstration of the XGB can be found in (c) [30d and dam] and (d) [180d and dam]. The feature importance of the lightGBM is demonstrated in (e) [30d and dam] and (f) [180d and dam]. The x-axis indicates the feature values. The factors classified in Fig. S3 are as follows: blue bars, components negatively associated with OCT to dam_H in Fig. S3; red bars, components positively associated with OCT to dam_H in Fig. S3. Abbreviations indicate as follows: D_, bacteria derived from dams; c_, bacteria derived from calves; and p_, plasma indices.

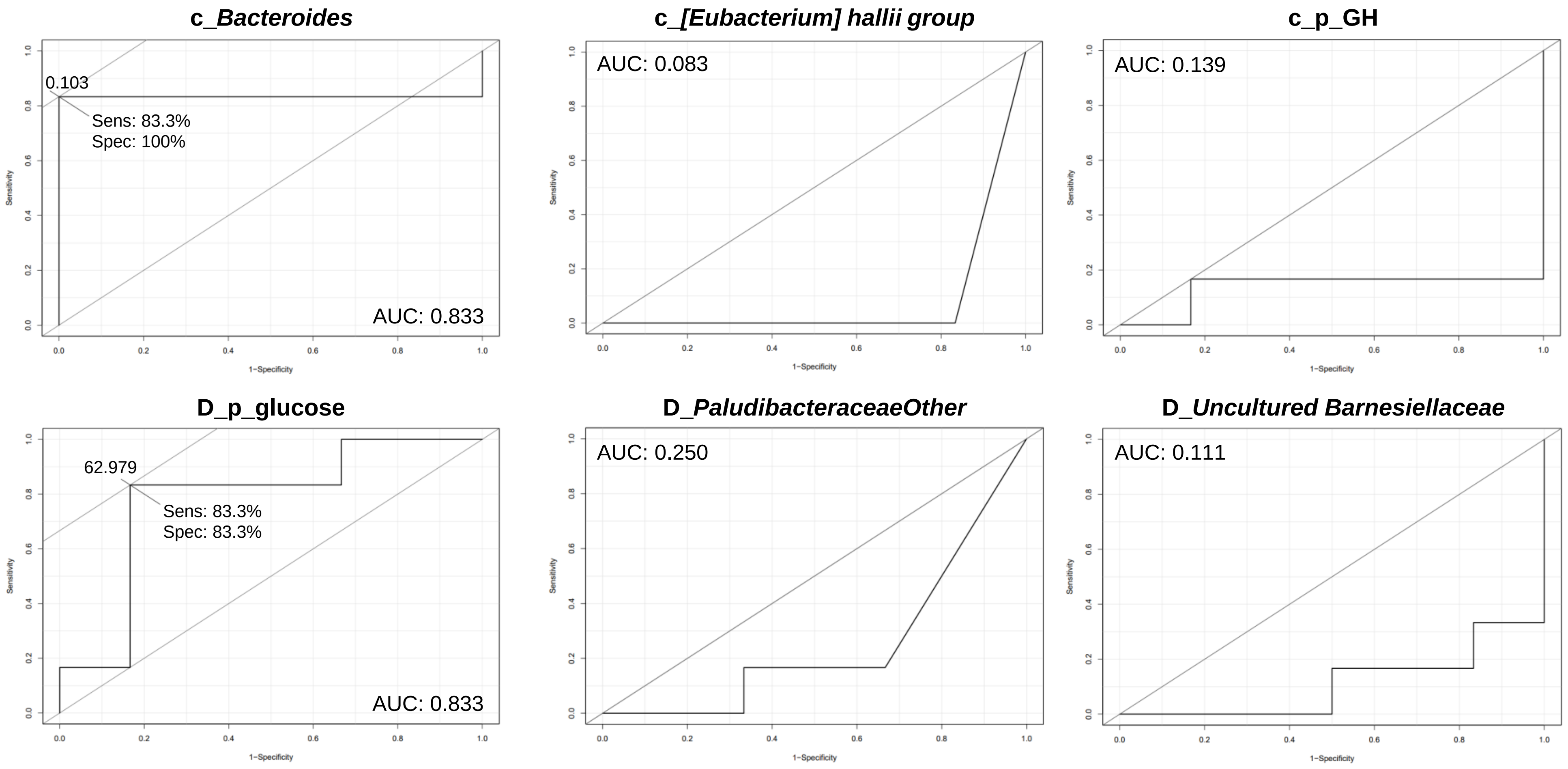

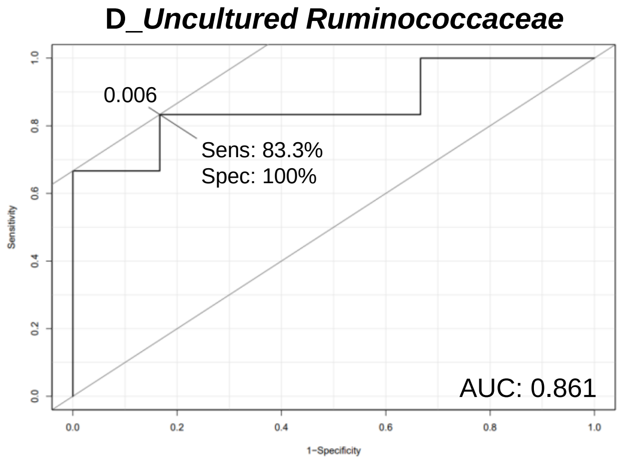

Fig. S6.

Receiver operating characteristic (ROC) curve analysis for ML-selected feature factors in Fig. 5a.

Accuracy evaluation of the feature selection of physical indices and bacterial genera of dams and their calves at 30d was calculated via ROC curve analysis. Abbreviations were as follows: Sensitivity, true positive rate (TPR); 1-Specificity, false positive rate (FPR); Sens, sensitivity; Spec, specificity; AUC, the area under the curve; D_, bacteria derived from dams; c_, bacteria derived from calves; p_, plasma indices; and GH, growth hormone.

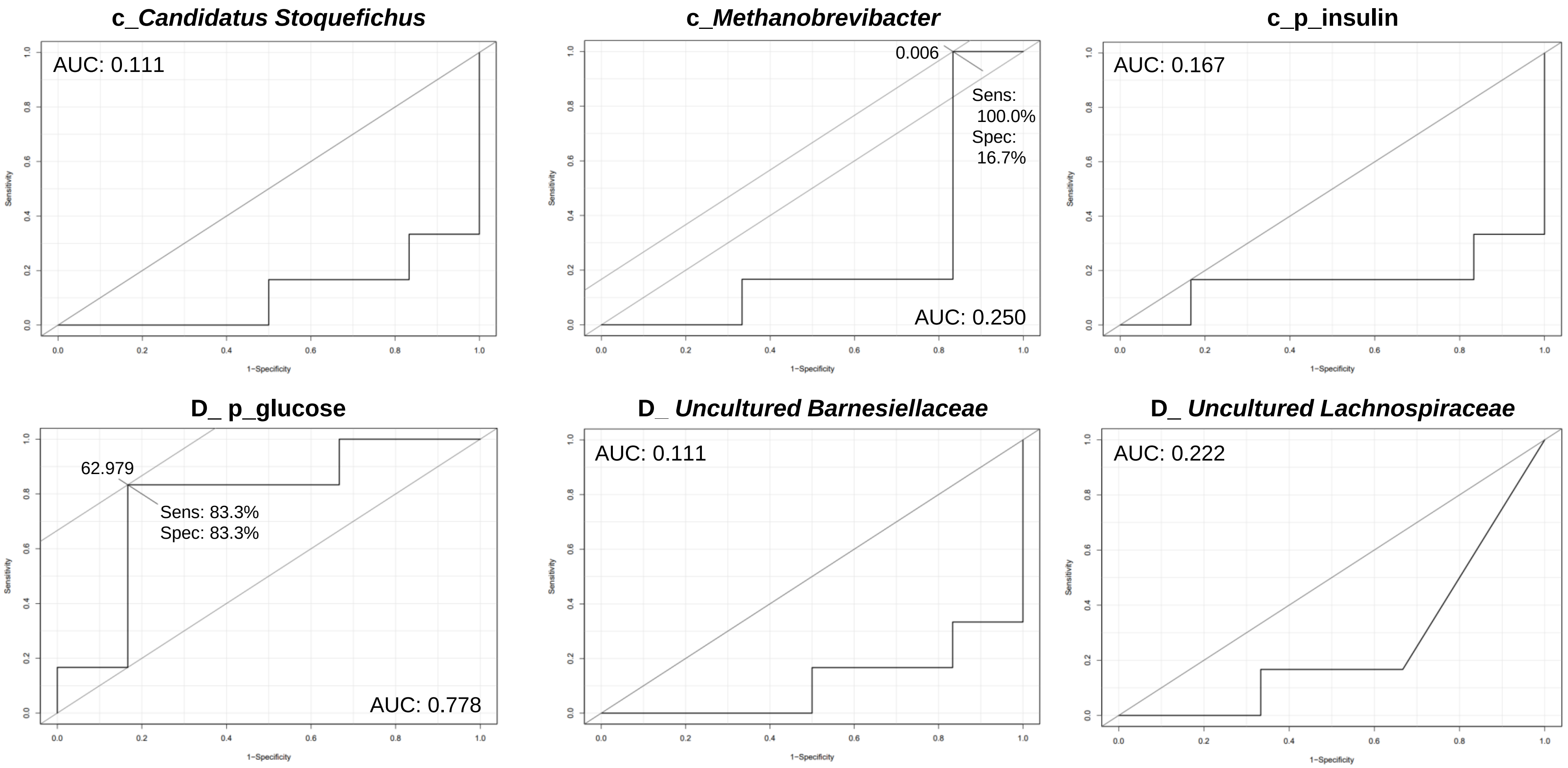

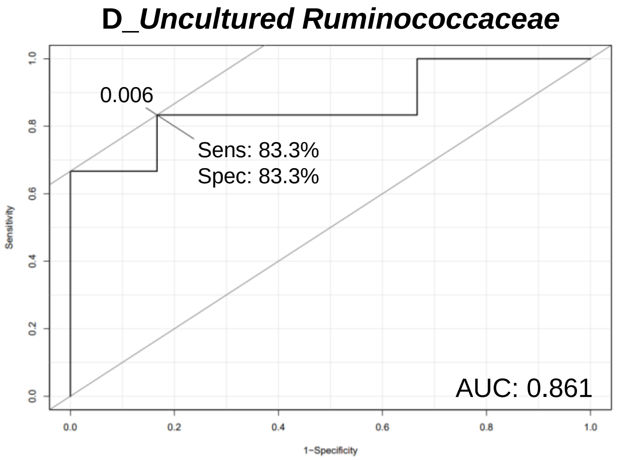

Fig. S7.

Receiver operating characteristic (ROC) curve analysis for ML-selected feature factors in Fig. 5b

Accuracy evaluation of the feature selection of physical indices and bacterial genera of dams and their calves at 30d was calculated via ROC curve analysis. Abbreviations were as follows: Sensitivity, true positive rate (TPR); 1-Specificity, false positive rate (FPR); Sens, sensitivity; Spec, specificity; AUC, the area under the curve; D_, bacteria derived from dams; c_, bacteria derived from calves; and p_, plasma indices.

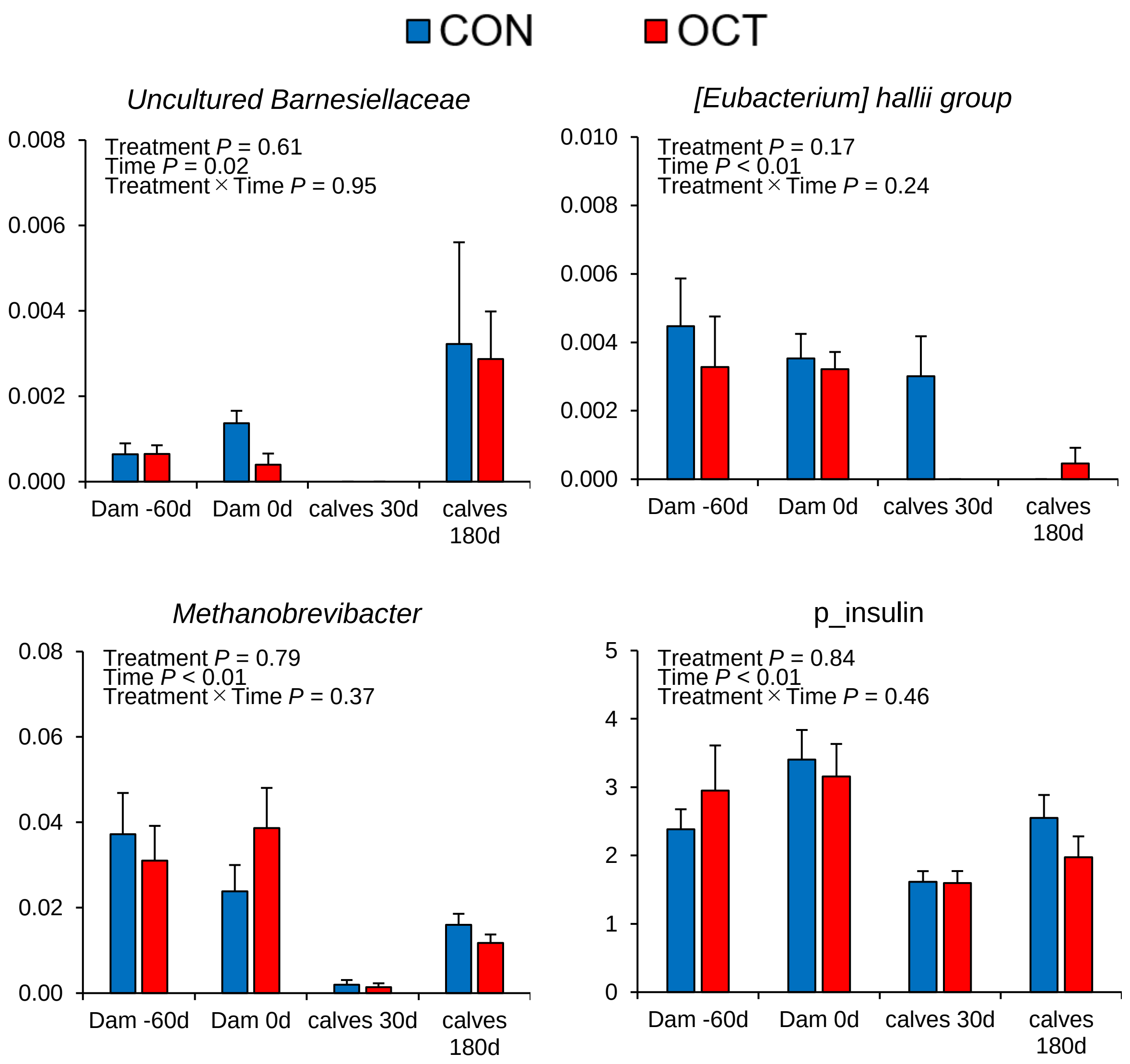

Fig. S8.

Relative abundance of the fecal bacterial genera indirectly linked with "OCT_to_dam" in Fig. 6a.

Dams were fed concentrate without (CON) or with Ca-octanoate supplementation (OCT) and calves were born from their dams. Data are presented as means ± SEM.

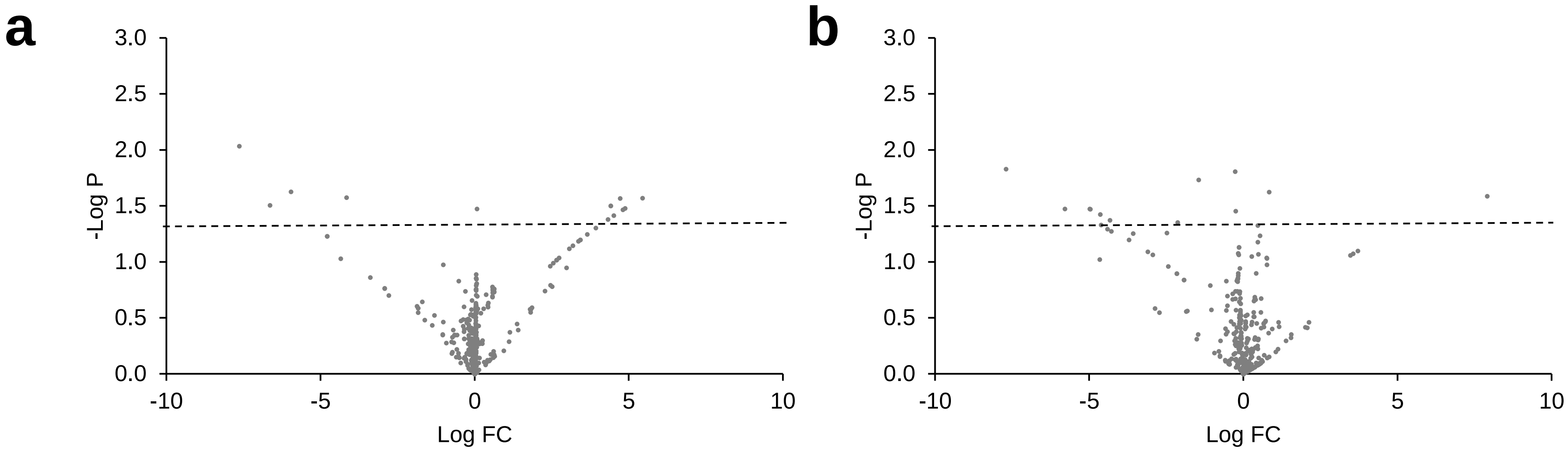

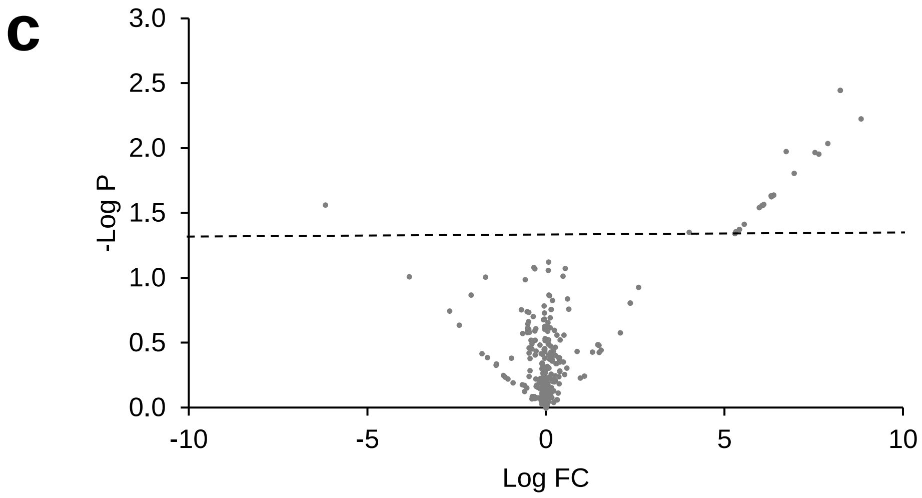

Fig. S9.

Volcano plot of pathways for dams and their calves.

Pathways were selected after preprocessing with a *P*-value adjusted cutoff of 0.05, as shown in (a) 0 d in dams, (b) 30 d in calves, and (c) 180 d in calves. No pathways were selected with significantly increased or decreased expression (P<0.05, false discovery rate (FDR)<0.1). The dotted lines indicate the *P*-value of 0.05.

Table S1. Growth performance of male and female calves born from dams fed concentrate without (CON) or with Ca-octanoate supplementation (OCT).

Item		Male		Female		*P*-value				
						Treatment		Time	Treatment × Time	
		CON (n=5)	OCT (n=3)	CON (n=1)	OCT (n=3)	Male	Female		Male	Female
BW, kg	birth	36.10 ± 2.25	36.17 ± 2.35	38.00 ± 0.00	29.83 ± 0.93	0.60	N.D.	<0.01	0.81	N.D.
	30d	53.20 ± 1.69	51.60 ± 2.27	53.73 ± 0.00	44.99 ± 3.55					
	180d	208.96 ± 5.43	204.33 ± 6.39	194.95 ± 0.00	170.32 ± 8.20					
ADG, kg/d	30d	0.57 ± 0.03	0.51 ± 0.02	0.52 ± 0.00	0.51 ± 0.09	0.36	N.D.	<0.01	0.56	N.D.
	180d	0.96 ± 0.03	0.93 ± 0.03	0.87 ± 0.00	0.78 ± 0.04					

Abbreviations indicate as follows: BW, body weight; ADG, average daily gain; and N.D., not determined.

Table S2. Feeding performance of male and female calves born from dams fed concentrate without (CON) or with Ca-octanoate supplementation (OCT).

	Male		Female		*P*-value	
Item	CON (n=5)	OCT (n=3)	CON (n=1)	OCT (n=3)	Male	Female
Preweaning Period (4-90d)						
MR intake, kg/d	0.90 ± 0.01	0.91 ± 0.01	0.92 ± 0.00	0.91 ± 0.01	0.46	N.D.
Starter intake, kg/d	0.55 ± 0.05	0.38 ± 0.03	0.27 ± 0.00	0.31 ± 0.06	0.06	N.D.
Hay intake, kg/d	0.50 ± 0.07	0.33 ± 0.07	0.32 ± 0.00	0.16 ± 0.03	0.19	N.D.
CP intake, kg/d	0.39 ± 0.01	0.35 ± 0.01	0.33 ± 0.00	0.32 ± 0.01	0.10	N.D.
EE intake, kg/d	0.18 ± 0.00	0.18 ± 0.00	0.17 ± 0.00	0.17 ± 0.00	0.32	N.D.
CF intake, kg/d	0.21 ± 0.03	0.15 ± 0.03	0.14 ± 0.00	0.08 ± 0.01	0.16	N.D.
G: F, kg/kg	0.49 ± 0.02	0.56 ± 0.03	0.52 ± 0.00	0.53 ± 0.05	0.05	N.D.
Postweaning Period (91-180d)						
Concentrate intake, kg/d	3.46 ± 0.08	3.66 ± 0.12	3.51 ± 0.00	2.98 ± 0.17	0.21	N.D.
Hay intake, kg/d	2.62 ± 0.15	1.99 ± 0.22	1.75 ± 0.00	1.72 ± 0.20	0.05	N.D.
CP intake, kg/d	0.73 ± 0.02	0.72 ± 0.01	0.68 ± 0.00	0.59 ± 0.03	0.74	N.D.
EE intake, kg/d	0.13 ± 0.00	0.12 ± 0.00	0.11 ± 0.00	0.10 ± 0.00	0.45	N.D.
CF intake, kg/d	1.28 ± 0.06	1.08 ± 0.07	0.98 ± 0.00	0.91 ± 0.07	0.07	N.D.
G: F, kg/kg	0.21 ± 0.01	0.21 ± 0.02	0.21 ± 0.00	0.21 ± 0.02	0.76	N.D.
Total Period, 4-180d						
CP intake, kg/d	0.56 ± 0.02	0.54 ± 0.01	0.51 ± 0.00	0.46 ± 0.02	0.32	N.D.
EE intake, kg/d	0.15 ± 0.00	0.15 ± 0.00	0.14 ± 0.00	0.14 ± 0.00	0.33	N.D.
CF intake, kg/d	0.76 ± 0.04	0.62 ± 0.05	0.57 ± 0.00	0.51 ± 0.04	0.08	N.D.
G: F, kg/kg	0.27 ± 0.01	0.29 ± 0.02	0.28 ± 0.00	0.29 ± 0.03	0.35	N.D.

Abbreviations indicate as follows: MR, milk replacer; CP, crude protein; EE, ether extract; CF, crude fiber; G: F, gain to feed intake ratio (Average daily gain per dry matter intake per day), and N.D., not determined.

Table S3. Statistical evaluation of the distribution of fecal bacteria and plasma indices data from the dams and their calves.

Item	*P*-value
D_*Uncultured Ruminococcaceae*_0d	0.1784
D_*Uncultured Barnesiellaceae*_0d	0.1303
D_*PaludibacteraceaeOther*_0d	0.0005
D_*Uncultured Lachnospiraceae*_0d	0.0016
D_p_glucose_0d	0.9682
c_*Bacteroides*_30d	0.9766
c_*Eubacterium_hallii_group*_30d	0.0007
c_p_GH_30d	0.0059
c_*Methanobrevibacter*_180d	0.4438
c_*Candidatus_Stoquefichus*_180d	0.0027
c_p_insulin_180d	0.0031

The normality of each bacteria and plasma indices selected by XGBoost analysis was speculated using Shapiro-wilk test.
